# General Relations between Stress Fluctuations and Viscoelasticity in Amorphous Polymer and Glass-Forming Systems

**DOI:** 10.3390/polym16162336

**Published:** 2024-08-18

**Authors:** Alexander Semenov, Jörg Baschnagel

**Affiliations:** Institut Charles Sadron, CNRS–UPR 22, University of Strasbourg, 67034 Strasbourg, France

**Keywords:** supercooled liquids, polymers, viscoelasticity, amorphous solids

## Abstract

Mechanical stress governs the dynamics of viscoelastic polymer systems and supercooled glass-forming fluids. It was recently established that liquids with long terminal relaxation times are characterized by transiently frozen stress fields, which, moreover, exhibit long-range correlations contributing to the dynamically heterogeneous nature of such systems. Recent studies show that stress correlations and relaxation elastic moduli are intimately related in isotropic viscoelastic systems. However, the origin of these relations (involving spatially resolved material relaxation functions) is non-trivial: some relations are based on the fluctuation-dissipation theorem (FDT), while others involve approximations. Generalizing our recent results on 2D systems, we here rigorously derive three exact FDT relations (already established in our recent investigations and, partially, in classical studies) between spatio-temporal stress correlations and generalized relaxation moduli, and a couple of new exact relations. We also derive several new approximate relations valid in the hydrodynamic regime, taking into account the effects of thermal conductivity and composition fluctuations for arbitrary space dimension. One approximate relation was heuristically obtained in our previous studies and verified using our extended simulation data on two-dimensional (2D) glass-forming systems. As a result, we provide the means to obtain, in any spatial dimension, all stress-correlation functions in terms of relaxation moduli and vice versa. The new approximate relations are tested using simulation data on 2D systems of polydisperse Lennard–Jones particles.

## 1. Introduction

Viscoelastic liquids and amorphous materials are characterized by long-lasting memory effects often involving a wide spectrum of relaxation times correlating the flow to the prior external forces and strains [1,2,3,4]. Examples of such materials include complex fluids like viscoelastic polymer melts and solutions, molten metallic alloys, glass-forming (supercooled) liquids and soft-matter systems [3,4,5,6,7,8,9,10,11,12,13,14,15,16,17,18,19]. The central physical quantity governing the dynamics of such materials is the mechanical stress [20]. Stress-correlation functions can provide important information on the rheological properties of amorphous systems, including the most important rheological functions like shear and longitudinal relaxation moduli (and the corresponding dynamical moduli) [3,4,21]. Moreover, glass-forming liquids are known to be highly heterogeneous (near or below the glass transition temperature $T_{g}$) [22,23,24,25,26,27,28,29], leading to a significant wave-vector dependence of their shear viscosity and relaxation moduli [30,31,32]. A similar behavior was observed [33] and predicted [34] for polymer liquids, and it is expected to be even more important for high-molecular-weight polymers.

Useful relations between the spatio-temporal stress correlation functions and the generalized (length-scale dependent) relaxation moduli (GRMs) have recently been obtained using the Zwanzig–Mori projection operator formalism [35,36] and the fluctuation-dissipation theorem (FDT) [32,37]. Based on these theoretical relations, it was established that liquids with long terminal relaxation times are characterized by transiently frozen stress fields, which, moreover, exhibit long-range correlations supporting the dynamically heterogeneous nature of glass-forming systems [32,35,36,37,38,39]. These theoretical predictions reinforce the conclusions of the prior extensive and pioneering simulation studies on stress-correlations in supercooled liquids [40,41,42,43] and also agree with more recent simulation results [32,44,45].

While the recent theoretical studies show that stress correlations and viscoelastic relaxation moduli are intimately related in complex fluids like polymer and supercooled liquids [32,35,36,37], the origin of these relations (involving spatially resolved relaxation functions) appears to be non-trivial: a number of relations are exact and follow from the fluctuation-dissipation theorem (FDT), while alternative physical arguments are required to derive other relations [32,37]. Generalizing our recent results on two-dimensional (2D) systems [32,37], we here obtain and discuss the full set of such stress–fluctuation relations valid for arbitrary space dimension.

In the next two sections, we reprise the relevant classical results on the bulk elastic and viscoelastic properties of amorphous systems, presenting the fully tensorial relations between fluctuations of volume-averaged stress and elastic (relaxation) moduli. The bulk equations are then generalized in Section 4 to deal with wave-vector $(\underline{q}$)-dependent stress correlations (characterized by the tensorial correlation function $C=C(\underline{q},t)$) and spatially resolved relaxation moduli (elasticity tensor $E=E(\underline{q},t)$). The methodologically new point here is that we first present a detailed derivation of the general *tensorial* equation linking *C*- and *E*-tensor fields, which then yields three basic relations between the generalized shear, longitudinal and transverse (mixed) relaxation moduli (G(q,t), L(q,t) and M(q,t)) on the one hand, and the invariant correlation functions on the other hand. The recently discovered *M*-relation (Equation (64)) then follows from the general tensorial equation in exactly the same way as other relations (Equations (62) and (63)). Different aspects concerning the definition of the *q*-dependent elasticity tensor *E* are discussed in Section 5. In particular, it is highlighted there that not all the components of *E* can be unambiguously defined for q≠0 based on a stress-to-strain response. In Section 6, we introduce the concept of stress noise $\sigma^{n}$ and propose a new definition of all components of the elasticity tensor $E(\underline{q},t)$ in terms of $\sigma^{n}$. It is also demonstrated there that the new definition is consistent with all the known properties of this tensor. On this basis, we derive the full set of exact relations between the correlation and elasticity tensors, $C(\underline{q},t)$ and $E(\underline{q},t)$, and establish two approximate relations allowing to obtain the full correlation tensor $C(\underline{q},t)$ in terms of only three material functions, G(q,t), L(q,t) and M(q,t), also known as viscoelastic memory functions (VMFs). We also discuss how to improve the accuracy of an approximate relation for two-dimensional systems. The theoretical predictions are then compared with simulation results on 2D polydisperse systems of Lennard–Jones (LJ) particles. Such 2D systems have been recently studied experimentally [46,47,48] and have received a lot of attention in simulation studies [32,44]. The main results of the paper are summarized in the last Section 7. In particular, the most important novel results are highlighted in the last point 11 of this section.

## 2. Classical Elasticity

For tutorial purposes, we start with the linear (affine) elasticity of a macroscopically uniform isotropic solid body. Its deformation is defined by the (coarse-grained) field of displacements $\underline{u}=\underline{u}\left(\underline{r}\right)$ of its material elements (here, $\underline{r}$ is the initial position of an element). At equilibrium ($\underline{u}=0$), the mean mechanical stress $\underline{\underline{\sigma }}={\underline{\underline{\sigma }}}^{\left(0\right)}$ is isotropic, $\sigma_{\alpha \beta }^{\left(0\right)}=-p_{0}\delta_{\alpha \beta }$, where $p_{0}$ is the external pressure [49]. (Note that in computer simulations the mean stress tensor for a given configuration can be slightly anisotropic. In this case, ${\underline{\underline{\sigma }}}^{\left(0\right)}$ should be considered as the mean stress tensor averaged over a sufficiently large ensemble of configurations). Let us consider a weak affine deformation

(1) $$\gamma_{\alpha \beta }\equiv \partial u_{\alpha }/\partial r_{\beta }$$

where $\gamma_{\alpha \beta }$ is the strain tensor which does not change in time, and α, β refer to Cartesian components. It leads to a macroscopically homogeneous stress as a linear response, $\Delta \sigma_{\alpha \beta }=\sigma_{\alpha \beta }-\sigma_{\alpha \beta }^{\left(0\right)}$, where $\sigma_{\alpha \beta }$ is the mean (time-averaged) stress in the deformed state. According to Hooke’s law

(2) $$\Delta \sigma_{\alpha \beta }=E_{\alpha \beta \alpha^{\prime }\beta^{\prime }}\gamma_{\alpha^{\prime }\beta^{\prime }}$$

where $E_{\alpha \beta \alpha^{\prime }\beta^{\prime }}$ is the tensor of static elastic moduli. (Here and below, the Einstein convention for summation over repeated indices is assumed). For systems (liquids) with no orientational order, the stress tensor is always symmetric, leading to

(3) $$E_{\alpha \beta \alpha^{\prime }\beta^{\prime }}=E_{\beta \alpha \alpha^{\prime }\beta^{\prime }}$$

To exclude a rotation of the system as a whole (which does not cost any energy), we can consider the symmetric part of $\underline{\underline{\gamma }}$,

(4) $$\epsilon_{\alpha \beta }=\frac{1}{2}\left(\gamma_{\alpha \beta }+\gamma_{\beta \alpha }\right)$$

The symmetric $\epsilon_{\alpha \beta }$ will be referred to as the classical strain. Then, Equation (2) transforms into the classical relation

(5) $$\Delta \sigma_{\alpha \beta }=E_{\alpha \beta \alpha^{\prime }\beta^{\prime }}\;\epsilon_{\alpha^{\prime }\beta^{\prime }}$$

The reason for the equivalence of the two equations, (2) and (5), is the minor symmetry of the *E*-tensor:

(6) $$E_{\alpha \beta \alpha^{\prime }\beta^{\prime }}=E_{\alpha \beta \beta^{\prime }\alpha^{\prime }}$$

coming from the assumed isotropy of the system demanding that *E* must be an isotropic tensor [44], which, together with the symmetry relation (3), leads to a well-known equation

(7) $$E_{\alpha \beta \alpha^{\prime }\beta^{\prime }}=\lambda \delta_{\alpha \beta }\delta_{\alpha^{\prime }\beta^{\prime }}+\mu \left(\delta_{\alpha \alpha^{\prime }}\delta_{\beta \beta^{\prime }}+\delta_{\alpha \beta^{\prime }}\delta_{\alpha^{\prime }\beta }\right)$$

with λ and μ being the Lamé coefficients [20,44,49]. (Note that Equation (6) also comes directly from Equation (2) since rotations of the body as a whole (corresponding to an anti-symmetric $\underline{\underline{\gamma }}$) must not lead to any change in the mean (ensemble-averaged) stress. The usefulness (convenience) of Equation (2) is also clarified in Section 5.3 in relation to the wave-vector- dependent elasticity).

The free energy increment ΔF associated with a small strain $\underline{\underline{\epsilon }}$ is

(8) $$\Delta F≃\frac{V}{2}E_{\alpha \beta \alpha^{\prime }\beta^{\prime }}\epsilon_{\alpha \beta }\epsilon_{\alpha^{\prime }\beta^{\prime }}$$

where *V* is the system volume and the higher-order terms in $\underline{\underline{\epsilon }}$ are omitted. (Note that Equation (8) remains valid also if $\epsilon_{\alpha \beta }$ is replaced with $\gamma_{\alpha \beta }$). Suppose the elastic body has a free surface, so it can be deformed by a thermal fluctuation. Then, by virtue of the Boltzmann equipartition principle Equation (8), it leads to the following correlation properties for thermal fluctuations of the classical strain, $\underline{\underline{\epsilon }}$:

(9) $$\langle \epsilon_{\alpha \beta }\epsilon_{\alpha^{\prime }\beta^{\prime }}\rangle =\frac{T}{4\mu V}\left[\delta_{\alpha \alpha^{\prime }}\delta_{\beta \beta^{\prime }}+\delta_{\alpha \beta^{\prime }}\delta_{\alpha^{\prime }\beta }-\frac{2\lambda }{2\mu +\lambda d}\delta_{\alpha \beta }\delta_{\alpha^{\prime }\beta^{\prime }}\right]$$

where $\epsilon_{\alpha \beta }$ and $\epsilon_{\alpha^{\prime }\beta^{\prime }}$ are strain components taken at the same time, the brackets $\langle ..\rangle$ mean the complete ensemble- and time-averaging, *d* is the space dimension, and *T* is the temperature in energy units.

It is also possible to relate stress fluctuations with the *E*-tensor. Here, it is useful to recall that the latter tensor reflects the long-time (static) stress response (cf Equation (2)); hence, the relevant stress correlation function

$$C_{\alpha \beta \alpha^{\prime }\beta^{\prime }}\equiv \frac{V}{T}\langle \delta \sigma_{\alpha \beta }\delta \sigma_{\alpha^{\prime }\beta^{\prime }}\rangle ,\;\delta \sigma_{\alpha \beta }\equiv \sigma_{\alpha \beta }-\langle \sigma_{\alpha \beta }\rangle$$

must involve a *quasi-static* (rather than instantaneous) stress $\sigma_{\alpha \beta }$ (note that $\langle \sigma_{\alpha \beta }\rangle =\sigma_{\alpha \beta }^{\left(0\right)}$). To define it, one has to assume that the strain fluctuations are sufficiently slow (cf refs. [50,51]) as compared to the internal structural relaxation of the system (with terminal relaxation time τ); the characteristic time of the strain fluctuations, $\tau_{strain}$, must be much longer than τ [50]. Therefore, $\sigma_{\alpha \beta }$ (involved in the definition of the *C*-tensor) must be considered as the stress component coarse-grained over a time interval Δt such that $\tau \ll \Delta t\ll \tau_{strain}$. In this case,

$$\delta \sigma_{\alpha \beta }≃E_{\alpha \beta \alpha^{\prime }\beta^{\prime }}\;\epsilon_{\alpha^{\prime }\beta^{\prime }}$$

(note that ϵ is a slow strain fluctuation, hence δσ, being a stress response to ϵ, cf Equation (5), is a fluctuation as well; that is why we use the notation δσ instead of Δσ here) so that using Equations (7) and (9), we obtain

(10) $$C_{\alpha \beta \alpha^{\prime }\beta^{\prime }}≃E_{\alpha \beta \alpha^{\prime }\beta^{\prime }}$$

Equation (9) can be obtained in a different way using the classical fluctuation-dissipation theorem (FDT) [52,53]. As before, one can define the mean strain, $\gamma_{\alpha \beta }=\left(1/V\right)\int \gamma_{\alpha \beta }\left(\underline{r}\right)d^{d}r$ (cf Equation (1)) based on the coarse-grained displacement field. The strain $\gamma_{\alpha \beta }$ can be considered as a tensorial variable conjugate to the external ‘force’, $\sigma_{\alpha \beta }^{ex}$, such that the external potential energy (the contribution of the ‘force’ to the free energy of the system) is

(11) $$U_{ext}=-V\gamma_{\alpha \beta }\sigma_{\alpha \beta }^{ex}$$

Obviously, the ‘force’ tensor $\sigma_{\alpha \beta }^{ex}$ is the external stress applied to the system [20]. The symmetric part of the deformation, $\epsilon_{\alpha \beta }$, induced by a *weak* external stress can be obtained by minimization of the total free energy $F_{tot}=\Delta F+U_{ext}$, where ΔF is defined in Equation (8). As a result, we obtain a relation between the mean deformation, $\langle \epsilon_{\alpha \beta }\rangle$, and $\sigma_{\alpha \beta }^{ex}$ (cf Equation (2)):

(12) $$E_{\alpha \beta \alpha^{\prime }\beta^{\prime }}\langle \epsilon_{\alpha^{\prime }\beta^{\prime }}\rangle =\sigma_{\alpha \beta }^{ex}$$

According to the classical FDT [52,53], the correlation tensor $\langle \epsilon_{\alpha \beta }\epsilon_{\alpha^{\prime }\beta^{\prime }}\rangle$ must be proportional to the susceptibility, $\partial \langle \epsilon_{\alpha^{\prime }\beta^{\prime }}\rangle /\partial \sigma_{\alpha \beta }^{ex}$:

(13) $$\langle \epsilon_{\alpha \beta }\epsilon_{\alpha^{\prime }\beta^{\prime }}\rangle =\frac{T}{V}\partial \langle \epsilon_{\alpha^{\prime }\beta^{\prime }}\rangle /\partial \sigma_{\alpha \beta }^{ex}$$

Equation (9) can then be deduced from Equations (7), (12) and (13).

## 3. Classical Viscoelasticity

Let us turn to viscoelastic systems, including polymer melts and solutions and glass-forming supercooled liquids, but also, in principle, glassy amorphous solids below the glass transition temperature $T_{g}$. Such systems are characterized by time-dependent relaxation moduli, like the shear relaxation modulus G(t). The goal is to find relations between the stress-correlation functions and the relaxation moduli. Obviously, the argument leading to Equation (10) is not applicable in this case due to its static nature. By contrast, it is well-known that the classical FDT can be applied to relaxation processes [52,53]. There is, however, a fundamental problem with its application to flows of liquids using the deformation tensor as a variable, which is based on the concept of a necessarily continuous (coarse-grained) displacement field $\underline{u}\left(\underline{r}\right)$, cf Equation (1). The point is that during long relaxation times characteristic of most viscoelastic liquids, the initially neighboring particles can go far away from each other (by self-diffusion), which means that $\underline{u}\left(\underline{r}\right)$ becomes ill-defined (virtually discontinuous). In other words, here, we arrive at a contradiction between continuum-field and corpuscular views on the fluid dynamics [20]. To avoid such problems, another version of the FDT [32,34,37] should be employed here. It is outlined below. (Note that this approach is applicable more generally also for networks with transient or permanent bonds and solid amorphous systems where $\underline{u}\left(\underline{r}\right)$ is well defined).

(i) We use a more precise definition of the elastic moduli. The tensor of relaxation moduli $E_{\alpha \beta \alpha^{\prime }\beta^{\prime }}\left(t\right)$ is defined via the stress tensor response, $\Delta \sigma_{\alpha \beta }\left(t\right)$, to a small but instantaneous deformation of the system, $\gamma_{\alpha \beta }$, at t=0:

(14) $$\Delta \sigma_{\alpha \beta }\left(t\right)=E_{\alpha \beta \alpha^{\prime }\beta^{\prime }}\left(t\right)\gamma_{\alpha^{\prime }\beta^{\prime }}$$

(cf Equation (2); note that $\Delta \sigma_{\alpha \beta }\left(t\right)$ is the mean, ensemble-averaged, stress increment at time *t*). This deformation must be *affine-canonical* [54,55,56]. (The transformation is necessarily canonical since we assume that the system dynamics remains Hamiltonian also with the perturbation), which implies changes of both the coordinates $(\underline{r}$) and the velocities $(\underline{v}$) of all particles:

(15) $$r_{\alpha }\to r_{\alpha }+\gamma_{\alpha \beta }r_{\beta },\;\;v_{\alpha }\to v_{\alpha }-\gamma_{\beta \alpha }v_{\beta }\;\;\mathrm{at}t=0$$

(ii) We assume that before the perturbation (at t<0), the system was at equilibrium, being characterized by an isothermal-isobaric distribution in the phase space:

(16) $$P\left(\Gamma \right)=P_{0}\left(\Gamma \right)=const\;e^{-H(\Gamma )/T}$$

where P is the probability density, and Γ stands for the microstate in the phase space (coordinates and velocities of all particles), $H\left(\Gamma \right)=H_{0}\left(\Gamma \right)+p_{0}V$, $H_{0}\left(\Gamma \right)$ is the system Hamiltonian, V=V(Γ), its volume, and $p_{0}$, the imposed pressure. (Note the normalization condition: $\int_{\Gamma }P_{0}\left(\Gamma \right)=1$.) Importantly, we consider a liquid or a *fully equilibrated* amorphous system here, so that their shape variations, which are allowed, do not bring about any correction to the Hamiltonian; the external stress corresponds solely to an *isotropic* pressure $p_{0}$.

Right after the instantaneous deformation (at $t=0^{+}$), the distribution changes to

(17) $$P\left(\Gamma \right)=P_{0}\left(\Gamma \right)+\Delta P\left(\Gamma \right)$$

The microscopic definition of the stress tensor reads [57]

(18) $$\sigma_{\alpha \beta }\left(\Gamma \right)=\frac{1}{V}\sum_{i>j}u_{ij}^{\prime }\left(r\right)\frac{r_{\alpha }r_{\beta }}{r}-\frac{1}{V}\sum_{i}m_{i}v_{i\alpha }v_{i\beta }$$

where $u_{ij}\left(r\right)$ is the interaction energy of a pair of interacting particles (i,j), *r* is the distance between them, $\underline{r}$ is the corresponding displacement vector (from *i* to *j*), $m_{i}$ is the mass of particle *i*, and $v_{i\alpha }$ is the α-component of its velocity. Using Equation (18), we find that the change in H(Γ) generated by the transformation of Equation (15) (for a system initially in microstate Γ) is

(19) $$\Delta H=V\delta \sigma_{\alpha \beta }\left(\Gamma \right)\gamma_{\alpha \beta }$$

where $\delta \sigma_{\alpha \beta }\left(\Gamma \right)=\sigma_{\alpha \beta }\left(\Gamma \right)-\langle \sigma_{\alpha \beta }\rangle =\sigma_{\alpha \beta }\left(\Gamma \right)+p_{0}\delta_{\alpha \beta }$ is the stress fluctuation, and $\langle \sigma_{\alpha \beta }\rangle$ is the equilibrium ensemble-averaged stress, $\langle \sigma_{\alpha \beta }\rangle =-p_{0}\delta_{\alpha \beta }$. Taking also into account that the transformation conserves the phase space, we obtain

(20) $$\Delta P\left(\Gamma \right)/P_{0}\left(\Gamma \right)=\frac{\Delta H}{T}=\frac{V}{T}\delta \sigma_{\alpha \beta }\left(\Gamma \right)\gamma_{\alpha \beta }$$

(iii) From the above equations, it immediately follows that

(21) $$\Delta \sigma_{\alpha \beta }\left(t\right)=\int_{\Gamma }\sigma_{\alpha \beta }\left(t|\Gamma \right)\Delta P\left(\Gamma \right)=\gamma_{\alpha^{\prime }\beta^{\prime }}\int_{\Gamma }\frac{V}{T}\sigma_{\alpha \beta }\left(t|\Gamma \right)P_{0}\left(\Gamma \right)\delta \sigma_{\alpha^{\prime }\beta^{\prime }}\left(\Gamma \right)$$

where $\sigma_{\alpha \beta }\left(t|\Gamma \right)$ is the stress at time *t* under the condition that at t=0 the system was in the microstate Γ, and $\Delta \sigma_{\alpha \beta }\left(t\right)=\langle \sigma_{\alpha \beta }\left(t\right)-\sigma_{\alpha \beta }\left(0^{-}\right)\rangle$ is the ensemble-averaged stress increment due to the deformation (recall that at t<0, the system was assumed to be fully equilibrated; hence, $\langle \sigma_{\alpha \beta }\left(0^{-}\right)\rangle =-p_{0}\delta_{\alpha \beta }$). Note that after the deformation, at t>0, the volume and shape of a system are not allowed to vary. Thus, e.g., the volume varies only within the ensemble, but not in time.

The last integral in Equation (21) is obviously equal to the stress correlation function

(22) $$C_{\alpha \beta \alpha^{\prime }\beta^{\prime }}\left(t\right)\equiv \frac{V}{T}\langle \delta \sigma_{\alpha \beta }\left(t\right)\delta \sigma_{\alpha^{\prime }\beta^{\prime }}\left(0\right)\rangle$$

where ‘0’ means t=0. On using the above equation and Equations (14) and (21), we find the FDT relation:

(23) $$E_{\alpha \beta \alpha^{\prime }\beta^{\prime }}\left(t\right)=C_{\alpha \beta \alpha^{\prime }\beta^{\prime }}\left(t\right)$$

It is important to note that, strictly speaking, Equation (23) is valid for a perfectly equilibrated isothermal-isobaric ensemble (as reflected in its probability distribution in the phase space at any instant before the perturbation) of either liquid or fully equilibrated amorphous solid systems whose *equilibrium* shear modulus $G_{e}$ is vanishing, $G_{e}=0$. (Note that $G_{e}=0$ does not exclude that the static modulus μ is positive since, in the case of an amorphous glassy system, μ corresponds to the long-time glassy plateau of G(t), which, however, eventually relaxes to 0 at t→∞.)

It is also noteworthy that the condition of perfect equilibration still allows for dynamical fluctuations of energy for each *individual system*, including the case of no such fluctuations, ie an energy-conserving and isochoric dynamics for each system (perhaps also involving periodic boundary conditions useful for simulations) [58,59]. In the general case, including isotropic liquids in the canonical or microcanonical isochoric ensembles, systems with canonical (Nosé–Hoover) or non-canonical (Gaussian isokinetic) thermostats, and amorphous systems equilibrated in a glassy state (a metabasin), a constant (time-independent) tensor must be added on the rhs of Equation (23) [51,58,59].

For isotropic systems, the general structure of $E_{\alpha \beta \alpha^{\prime }\beta^{\prime }}\left(t\right)$ is analogous to the static Equation (7):

(24) $$E_{\alpha \beta \alpha^{\prime }\beta^{\prime }}\left(t\right)=M\left(t\right)\delta_{\alpha \beta }\delta_{\alpha^{\prime }\beta^{\prime }}+G\left(t\right)\left(\delta_{\alpha \alpha^{\prime }}\delta_{\beta \beta^{\prime }}+\delta_{\alpha \beta^{\prime }}\delta_{\alpha^{\prime }\beta }\right)$$

where M(t) and G(t) are the generalized Lamé coefficients, λ and μ, respectively. At long times, in the quasi-static regime, $t>\tau_{s}$, where the relaxation moduli change weakly or vanish (note that at $T<T_{g}$, this regime corresponds to the glassy plateau), Equation (24) provides the static response in agreement with Equation (7): $M(t>\tau_{s})\approx \lambda$, $G(t>\tau_{s})\approx \mu$.

Note that in the general case, the stress response depends on whether the deformation was isothermic, adiabatic or, else, an imperfect control of temperature is involved (in numerical studies, it may correspond to an isokinetic thermostat, energy-conserving microcanonical simulation, and Nose–Hoover thermostatting, respectively). The thermostatting issues do not affect the shear modulus G(t), but can be important for M(t) [32,60]. Note also that the instantaneous response reflected in the affine moduli, G(0) and M(0), is always adiabatic (unless a perfect thermostatting of the system is provided) [58].

The above approach can be used to find an increment of any variable X=X(Γ) upon the affine-canonical deformation. The result is

(25) $$\Delta X\left(t\right)=\frac{1}{T}\langle X(t)\Delta H\rangle =\frac{V}{T}\gamma_{\alpha \beta }\langle X\left(t\right)\delta \sigma_{\alpha \beta }\left(0\right)\rangle ,\;t>0.$$

The variable X(t) in the rhs of the above equation can be replaced with δX(t) since $\langle \delta \sigma_{\alpha \beta }\left(0\right)\rangle =0$ by definition ($\delta \sigma_{\alpha \beta }=\sigma_{\alpha \beta }-\langle \sigma_{\alpha \beta }\rangle$, see text below Equation (19)).

## 4. Space-Resolved Viscoelasticity

In the previous section, we considered classical relaxation moduli related to a response of the volume-averaged stress to a perturbative affine deformation. Let us turn to the space-resolved viscoelasticity providing, in particular, a position-dependent response to a (possibly) localized perturbation. Inspired by the Boltzmann superposition principle [61], one can regard a weak continuous deformation of the system (e.g., in the course of its slow flow) as a superposition of small strains $d\gamma_{\alpha \beta }={\dot{\gamma }}_{\alpha \beta }dt$, where (cf Equation (1))

(26) $${\dot{\gamma }}_{\alpha \beta }=\frac{\partial v_{\alpha }}{\partial r_{\beta }}$$

is the strain rate and $\underline{v}$ is the flow velocity. Thus, generalizing Equation (14) (considering the stress field $\sigma_{\alpha \beta }\left(\underline{r},t\right)$ [20,62], instead of the volume-averaged stress, $\sigma_{\alpha \beta }\left(t\right)$), we can write (based on the superposition principle due to the adopted linear response approximation and recalling the space-time uniformity of the equilibrium macroscopically homogeneous systems we consider)

(27) $$\Delta \sigma_{\alpha \beta }\left(\underline{r},t\right)=\int_{0^{-}}^{t}{\tilde{E}}_{\alpha \beta \alpha^{\prime }\beta^{\prime }}\left(\underline{r}-{\underline{r}}^{\prime },t-t^{\prime }\right){\dot{\gamma }}_{\alpha^{\prime }\beta^{\prime }}\left({\underline{r}}^{\prime },t^{\prime }\right)dt^{\prime }d^{d}r^{\prime }$$

where $\Delta \sigma_{\alpha \beta }\left(\underline{r},t\right)$ is the ensemble-averaged stress response to the flow (which was absent at t<0). (A canonical ensemble is assumed by default in this section focused on space-resolved viscoelasticity for an arbitrary but finite wave-vector, $\underline{q}\neq 0$. By contrast to $\underline{q}=0$, which is sensitive to the thermodynamic boundary conditions (fixed volume or fixed pressure), all fluctuations at a finite $\underline{q}$ are disentangled from fluctuations of global variables like total energy or volume.) Note that the response relation, Equation (27), is different in nature from Equation (21) since the condition of no deformation (no flow) at t>0 was assumed in the relevant part of Section 3. Note that the response relation, Equation (27), is also different in nature from Equation (72) since in the latter case (of Equation (72)), the flow at t>0 is not prescribed in contrast to Equation (27), where ${\dot{\gamma }}_{\alpha^{\prime }\beta^{\prime }}\left({\underline{r}}^{\prime },t^{\prime }\right)$ is considered as a known field.

It is now instructive to provide a microscopic definition of the velocity field:

(28) $$\underline{v}\left(\underline{r},t\right)=\rho_{0}^{-1}\sum_{i=1}^{N}m_{i}{\underline{v}}_{i}\delta \left(\underline{r}-{\underline{r}}_{i}\right)$$

where $\rho_{0}=M/V$ is the mean mass density (M is the total mass of the system and *N* is the total number of particles), and ${\underline{r}}_{i}={\underline{r}}_{i}\left(t\right)$, ${\underline{v}}_{i}={\underline{v}}_{i}\left(t\right)$ are the position and velocity of particle *i*. Equation (27) can be rewritten in terms of Fourier transforms (FT) of the position-dependent functions. For example, the FT of $\tilde{E}$ is the wave-vector $(\underline{q}$)-dependent tensor of relaxation moduli:

(29) $$E_{\alpha \beta \alpha^{\prime }\beta^{\prime }}\left(\underline{q},t\right)\equiv \int {\tilde{E}}_{\alpha \beta \alpha^{\prime }\beta^{\prime }}\left(\underline{r},t\right)\exp\left(-i\underline{q}\cdot \underline{r}\right)d^{d}r$$

The FT of any other relevant function, $f(\underline{r})$ (where *f* may stay for a component of $\underline{\underline{\sigma }}$ or $\underline{\underline{\dot{\gamma }}}$ tensors) is generically defined as

(30) $$f\left(\underline{q}\right)=\frac{1}{V}\int f\left(\underline{r}\right)\exp\left(-i\underline{q}\cdot \underline{r}\right)d^{d}r$$

This way, $f(\underline{q})$ and $f(\underline{r}$) have the same physical dimension (note that here and below, we distinguish the original function from its FT by the argument only, $\underline{q}$ or $\underline{r}$). The inverse Fourier transform is given by

(31) $$f\left(\underline{r}\right)=\sum_{\underline{q}}f\left(\underline{q}\right)\exp\left(i\underline{q}\cdot \underline{r}\right)$$

where the sum runs over all $\underline{q}$-modes defined by the system size. In particular, the strain rate in Equation (27) is

$${\dot{\gamma }}_{\alpha \beta }\left(\underline{r},t\right)=\sum_{\underline{q}}{\dot{\gamma }}_{\alpha \beta }\left(\underline{q},t\right)\exp\left(i\underline{q}\cdot \underline{r}\right)$$

where (cf Equation (26))

(32) $${\dot{\gamma }}_{\alpha \beta }\left(\underline{q},t\right)=iv_{\alpha }\left(\underline{q},t\right)q_{\beta }$$

Thus, Equation (27) leads to

(33) $$\Delta \sigma_{\alpha \beta }\left(\underline{q},t\right)=\int_{0^{-}}^{t}E_{\alpha \beta \alpha^{\prime }\beta^{\prime }}\left(\underline{q},t-t^{\prime }\right){\dot{\gamma }}_{\alpha^{\prime }\beta^{\prime }}\left(\underline{q},t^{\prime }\right)dt^{\prime }$$

The kernel $E_{\alpha \beta \alpha^{\prime }\beta^{\prime }}$ here is the tensor of the generalized ($\underline{q}$-dependent) relaxation moduli. Equation (33) can be considered as a $\left(\underline{q},t\right)$-dependent generalization of Equation (2). As to why we use here the γ-strain instead of the classical symmetrized ϵ-strain, see Section 5.3.

Since physical variables like ${\underline{r}}_{a}$, ${\underline{v}}_{a}$, $\sigma_{\alpha \beta }\left(\underline{r}\right)$ are necessarily real, changing $\underline{q}$ to $-\underline{q}$ always leads to complex conjugation of a $\underline{q}$-dependent variable. Hence, for example, $\sigma_{\alpha \beta }\left(-\underline{q},t\right)=\sigma_{\alpha \beta }^{*}\left(\underline{q},t\right)$, where star (*) means complex conjugate (cf Equation (30)). Also, obviously, the tensor ${\tilde{E}}_{\alpha \beta \alpha^{\prime }\beta^{\prime }}\left(\underline{r},t\right)$ must be real and for all isotropic achiral systems, it is an isotropic tensor field [44], which (being a 4th-rank tensor) must be even in $\underline{r}$:

$${\tilde{E}}_{\alpha \beta \alpha^{\prime }\beta^{\prime }}\left(\underline{r},t\right)={\tilde{E}}_{\alpha \beta \alpha^{\prime }\beta^{\prime }}\left(-\underline{r},t\right)$$

Hence, by virtue of Equation (29), the same must be true for its Fourier transform:

(34) $$E_{\alpha \beta \alpha^{\prime }\beta^{\prime }}\left(\underline{q},t\right)=E_{\alpha \beta \alpha^{\prime }\beta^{\prime }}\left(-\underline{q},t\right)=E_{\alpha \beta \alpha^{\prime }\beta^{\prime }}^{*}\left(\underline{q},t\right)$$

Thus, the tensor $E_{\alpha \beta \alpha^{\prime }\beta^{\prime }}\left(\underline{q},t\right)$ is real; it is also obviously symmetric with respect to permutation of α and β (cf Equation (3)).

Note that for q=0 and ${\dot{\gamma }}_{\alpha \beta }\left(0,t^{\prime }\right)=\gamma_{\alpha \beta }\delta \left(t\right)$ corresponding to instantaneous affine deformation $\gamma_{\alpha \beta }$, Equation (33) becomes identical to Equation (14). It is also obvious that the velocity $\underline{v}\left(\underline{q},t\right)$ is proportional to the ‘current’ (momentum density) $\underline{J}$:

(35) $$\underline{v}\left(\underline{q},t\right)=\underline{J}\left(\underline{q},t\right)/\rho_{0}$$

where

(36) $$\underline{J}\left(\underline{q},t\right)=V^{-1}\sum_{i=1}^{N}m_{i}{\underline{v}}_{i}\left(t\right)\exp\left(-i\underline{q}\cdot {\underline{r}}_{i}\left(t\right)\right)$$

The current in the real space is

(37) $$\underline{J}\left(\underline{r},t\right)=\sum_{i}m_{i}{\underline{v}}_{i}\left(t\right)\delta \left(\underline{r}-{\underline{r}}_{i}\left(t\right)\right)$$

Our next step will be to accept Equation (33) (which is equivalent to Equation (27)) and to try and find a relation of its kernel *E* with the stress correlation functions. To this end, consider a system which was at equilibrium at t<0 (with no flow on the average)

$$\underline{v}=0\;\mathrm{at}t<0$$

but where the flow was generated at t≥0 by a perturbative external force field such that the force on particle *i* is

$${\underline{F}}_{i}\left(t\right)=m_{i}\underline{A}\left({\underline{r}}_{i}\left(t\right),t\right)$$

where $\underline{A}=\underline{A}\left(\underline{r},t\right)$ is a continuous vector field. Such forces $F_{i}$ provide a coherent acceleration $\underline{A}\left(\underline{r},t\right)$ of all particles. The external force density, therefore, is

(38) $$\underline{f}\left(\underline{r},t\right)=\underline{A}\left(\underline{r},t\right)\rho \left(\underline{r},t\right)$$

where

(39) $$\rho \left(\underline{r},t\right)=\sum_{i}m_{i}\delta \left(\underline{r}-{\underline{r}}_{i}\left(t\right)\right),\;\;\rho \left(\underline{q},t\right)=\frac{1}{V}\sum_{i=1}^{N}m_{i}\exp\left(-i\underline{q}\cdot {\underline{r}}_{i}\left(t\right)\right)$$

is the microscopic mass density.

To simplify the argument, let us consider a very short perturbation,

(40) $$\underline{A}\left(\underline{r},t\right)=\underline{V}\left(\underline{r}\right)\delta \left(t\right)$$

where $\underline{V}\left(\underline{r}\right)$ is the coherent velocity increment at position $\underline{r}$. Just like the rapid deformation considered in the previous section, this perturbation, $\underline{v}\to \underline{v}+\underline{V}\left(\underline{r}\right)$, must lead to a change in the distribution P(Γ) in the phase space, from the canonical $P_{0}\left(\Gamma \right)$ to $P_{0}\left(\Gamma \right)+\Delta P\left(\Gamma \right)$. As before, this transformation conserves the phase-space measure and leads (for a given initial microstate Γ) to the energy (H) increment

(41) $$\Delta H=\sum_{i}m_{i}{\underline{v}}_{i}\cdot \underline{V}\left({\underline{r}}_{i}\right)=\int \underline{J}\left(\underline{r},0\right)\cdot \underline{V}\left(\underline{r}\right)d^{d}r$$

Hence, the transformation leads to the following increment of a variable *X* at t>0 (cf Equation (25)):

(42) $$\Delta X\left(t\right)=\frac{1}{T}\langle X(t)\Delta H\rangle =\frac{1}{T}\int \langle X\left(t\right)\underline{J}\left(\underline{r},0\right)\rangle \cdot \underline{V}\left(\underline{r}\right)d^{d}r$$

where $\underline{J}\left(\underline{r},t\right)$ is defined in Equation (37). At this point, it is convenient to focus on just a single wave-vector $\underline{q}$ setting

(43) $$\underline{V}\left(\underline{r}\right)=\underline{V}\left(\underline{q}\right)\exp\left(i\underline{q}\cdot \underline{r}\right)$$

and choosing $X\left(t\right)=\sigma_{\alpha \beta }\left(\underline{q},t\right)$. (Note that $\underline{V}\left(\underline{q}\right)$ here is a constant vector.) Then, Equation (41) transforms to

(44) $$\Delta H=V\underline{J}\left(-\underline{q},0\right)\cdot \underline{V}(\underline{q)}$$

while Equation (42) reduces to

(45) $$\Delta \sigma_{\alpha \beta }\left(\underline{q},t\right)=C_{\alpha \beta \alpha^{\prime }}^{\sigma J}\left(\underline{q},t\right)V_{\alpha^{\prime }}\left(\underline{q}\right)$$

where

(46) $$C_{\alpha \beta \alpha^{\prime }}^{\sigma J}\left(\underline{q},t\right)\equiv \frac{V}{T}\langle \sigma_{\alpha \beta }\left(\underline{q},t+t^{\prime }\right)J_{\alpha^{\prime }}\left(-\underline{q},t^{\prime }\right)\rangle$$

is the cross-correlation function of the stress and current, which does not depend on $t^{\prime }$ since time is uniform (and we consider a stationary well-equilibrated system). Note that

(47) $$C_{\alpha \beta \alpha^{\prime }}^{\sigma J}\left(\underline{q},0\right)=0,\;\Delta \sigma_{\alpha \beta }\left(\underline{q},0\right)=0$$

due to time reversibility.

The function $C_{\alpha \beta \alpha^{\prime }}^{\sigma J}\left(\underline{q},t\right)$ is related to the generalized stress-correlation function (cf Equation (22))

(48) $$C_{\alpha \beta \alpha^{\prime }\beta^{\prime }}\left(\underline{q},t\right)\equiv \frac{V}{T}\langle \delta \sigma_{\alpha \beta }\left(\underline{q},t+t^{\prime }\right)\delta \sigma_{\alpha^{\prime }\beta^{\prime }}\left(-\underline{q},t^{\prime }\right)\rangle$$

To establish this relation, we employ the fundamental momentum equation [21,37]

$$\frac{\partial J_{\alpha }}{\partial t}=\frac{\partial \sigma_{\alpha \beta }}{\partial r_{\beta }}$$

which reads in Fourier space:

(49) $$\frac{\partial J_{\alpha }\left(\underline{q},t\right)}{\partial t}=iq_{\beta }\sigma_{\alpha \beta }\left(\underline{q},t\right)$$

Note that Equation (49) does not assume an ensemble averaging; it is valid microscopically for each system. On using Equations (46) and (48), it leads to

(50) $$\frac{\partial }{\partial t}C_{\alpha \beta \alpha^{\prime }}^{\sigma J}\left(\underline{q},t\right)=iC_{\alpha \beta \alpha^{\prime }\beta^{\prime }}\left(\underline{q},t\right)q_{\beta^{\prime }}$$

It is now convenient to deal with time-dependent functions, f(t), in terms of their modified Laplace transform (s-transform) [32,44]

(51) $$f\left(s\right)\equiv s\int_{0^{-}}^{\infty }f\left(t\right)e^{-st}dt$$

The transformed Equation (50) reads

(52) $$C_{\alpha \beta \alpha^{\prime }}^{\sigma J}\left(\underline{q},s\right)=\frac{i}{s}C_{\alpha \beta \alpha^{\prime }\beta^{\prime }}\left(\underline{q},s\right)q_{\beta^{\prime }}$$

so that Equation (45) leads to

(53) $$\Delta \sigma_{\alpha \beta }\left(\underline{q},s\right)=\frac{i}{s}C_{\alpha \beta \alpha^{\prime }\beta^{\prime }}\left(\underline{q},s\right)q_{\beta^{\prime }}V_{\alpha^{\prime }}(\underline{q)}$$

(Note that Equation (47) was taken into account here). Setting $X\left(t\right)=J_{\alpha }\left(\underline{q},t\right)$ in the general Equation, we obtain the response

(54) $$\Delta J_{\alpha }\left(\underline{q},s\right)=C_{\alpha \alpha^{\prime }}^{JJ}\left(\underline{q},s\right)V_{\alpha^{\prime }}\left(\underline{q}\right)$$

where

(55) $$C_{\alpha \alpha^{\prime }}^{JJ}\left(\underline{q},t\right)=\frac{V}{T}\langle J_{\alpha }\left(\underline{q},t+t^{\prime }\right)J_{\alpha^{\prime }}\left(-\underline{q},t^{\prime }\right)\rangle$$

is the current correlation function [21,57] whose *s*-transform, $C_{\alpha \alpha^{\prime }}^{JJ}\left(\underline{q},s\right)$, is related to the stress correlation function [37]:

(56) $$C_{\alpha \alpha^{\prime }}^{JJ}\left(\underline{q},s\right)=\rho_{0}\delta_{\alpha \alpha^{\prime }}-C_{\alpha \beta \alpha^{\prime }\beta^{\prime }}\left(\underline{q},s\right)q_{\beta }q_{\beta^{\prime }}/s^{2}$$

The above equation can be derived using the momentum equation just like Equation (52) (with the only difference that $C_{\alpha \alpha^{\prime }}^{JJ}\left(\underline{q},t=0\right)=\rho_{0}\delta_{\alpha \alpha^{\prime }}$ is nonzero since, as follows from Equations (36), (40) and (43), $\Delta \underline{J}\left(\underline{q},t=0^{+}\right)=\rho_{0}\underline{V}$$\left(\underline{q}\right)$).

The *s*-transform of Equation (33) reads:

(57) $$\Delta \sigma_{\alpha \beta }\left(\underline{q},s\right)=E_{\alpha \beta \alpha^{\prime }\beta^{\prime }}\left(\underline{q},s\right)\gamma_{\alpha^{\prime }\beta^{\prime }}\left(\underline{q},s\right)$$

where

(58) $$\gamma_{\alpha^{\prime }\beta^{\prime }}\left(\underline{q},s\right)=\frac{1}{s}{\dot{\gamma }}_{\alpha^{\prime }\beta^{\prime }}\left(\underline{q},s\right)=\frac{i}{s}\frac{1}{\rho_{0}}\Delta J_{\alpha^{\prime }}\left(\underline{q},s\right)q_{\beta^{\prime }}$$

since ${\dot{\gamma }}_{\alpha^{\prime }\beta^{\prime }}\left(\underline{q},t\right)=\left(i/\rho_{0}\right)J_{\alpha^{\prime }}\left(\underline{q},t\right)q_{\beta^{\prime }}$, as follows from Equations (32) and (35). Using Equations (53), (54) and (56)–(58) and taking into account that $\underline{V}\left(\underline{q}\right)$ is an arbitrary vector, we obtain

(59) $$C_{\alpha \beta \alpha^{\prime }\beta^{\prime }}\left(\underline{q},s\right)q_{\beta^{\prime }}=E_{\alpha \beta \alpha^{\prime }\beta^{\prime }}\left(\underline{q},s\right)q_{\beta^{\prime }}-\frac{E_{\alpha \beta \mu \beta^{\prime }}\left(\underline{q},s\right)q_{\beta^{\prime }}}{\rho_{0}s^{2}}C_{\mu \delta \alpha^{\prime }\delta^{\prime }}\left(\underline{q},s\right)q_{\delta }q_{\delta^{\prime }}$$

The above equation provides the general FDT relation between the stress correlation function *C* and the tensor *E* of the viscoelastic relaxation moduli. It can be simplified using the naturally rotated coordinate frame (NRC) [37,44] with axis 1 parallel to wave-vector $\underline{q}$:

(60) $$C_{\alpha \beta \gamma 1}\left(\underline{q},s\right)=E_{\alpha \beta \gamma 1}\left(\underline{q},s\right)-\frac{q^{2}}{\rho_{0}s^{2}}E_{\alpha \beta \mu 1}\left(\underline{q},s\right)C_{\mu 1\gamma 1}\left(\underline{q},s\right)$$

Equation (60) can be solved for $C_{\alpha \beta \gamma 1}\left(\underline{q},s\right)$ with a given $E_{\alpha \beta \gamma 1}\left(\underline{q},s\right)$ by first setting β=1 and then treating it as a standard matrix equation. Once $C_{\alpha 1\gamma 1}\left(\underline{q},s\right)$ is known, $C_{\alpha \beta \gamma 1}\left(\underline{q},s\right)$ can be obtained directly from Equation (60). As follows from the (αβ) symmetry of the tensor $E_{\alpha \beta \gamma 1}$ (which is obviously also applicable to the tensor $C_{\alpha \beta \gamma 1}$ [37]) and their invariance with respect to rotations around the main axis 1, there are only three independent components (involved in Equation (60)) in each tensor, $E_{\alpha \beta \gamma 1}\left(\underline{q},s\right)$ and $C_{\alpha \beta \gamma 1}\left(\underline{q},s\right)$. For the elasticity tensor, these components are

(61) $$L\left(q,s\right)\equiv E_{1111},G\left(q,s\right)\equiv E_{2121}\mathrm{and}M\left(q,s\right)\equiv E_{2211}$$

known as the longitudinal, shear and mixed (transverse) modulus, respectively (cf ref. [37]). (Another way to identify the three material functions, *L*, *G* and *M*, is expressed by Equation (79) in the next section.) Note that the functions *L*, *G* and *M* are all real and do not depend on the orientation of $\underline{q}$ (cf Equation (34) and ref. [37]). The relevant three general relations derived from Equation (60) (for q≠0) in the NRC are:

(62) $$C_{G}\left(q,s\right)\equiv C_{2121}\left(q,s\right)=\frac{G(q,s)}{1+G\left(q,s\right)q^{2}/\left(\rho_{0}s^{2}\right)}$$

(63) $$C_{L}\left(q,s\right)\equiv C_{1111}\left(q,s\right)=\frac{L(q,s)}{1+L\left(q,s\right)q^{2}/\left(\rho_{0}s^{2}\right)}$$

(64) $$C_{M}\left(q,s\right)\equiv C_{2211}\left(q,s\right)=\frac{M(q,s)}{1+L\left(q,s\right)q^{2}/\left(\rho_{0}s^{2}\right)}$$

These relations have already been stated in refs. [32,34,37,59]. The first Equation (62) is rather well known [57,63,64]. Noteworthily, the above relations are valid both for liquid systems (above the glass transition) and for amorphous solids (vitrified liquids), provided that they are completely equilibrated thermodynamically. Still, they are also valid for metastable glassy systems (trapped in a metabasin), provided that the lifetime of the metastable state is much longer than 1/s [32].

The three basic relations (62)–(64) allow to obtain all the three GRMs, G(q,t), L(q,t) and M(q,t), based on the stress-correlation functions. However, the reverse (to obtain all components of $C_{\alpha \beta \alpha^{\prime }\beta^{\prime }}\left(\underline{q},t\right)$ based on the moduli) is, strictly speaking, impossible. Nevertheless, it is still possible to approximately find the undefined components of $C_{\alpha \beta \alpha^{\prime }\beta^{\prime }}$ for small *q* using the three material functions. This is performed in Section 6.4. Furthermore, the basic Equation (59) is rederived and generalized in Section 6 using a different method (the mesoscopic approach involving the concept of stress noise). This way, we both demonstrate the consistency of our approaches and provide a framework for the approximate hydrodynamic theory.

The main results obtained above are discussed in the next section.

## 5. Preliminary Discussion

### 5.1. Applied Strain vs. External Force

Equation (33) can be converted to a rather standard definition of $E_{\alpha \beta \gamma \delta }\left(\underline{q},t\right)$ as a stress response, $\Delta \underline{\underline{\sigma }}=\Delta {\underline{\underline{\sigma }}}^{\left(r\right)}$, to a small instantaneous *q*-dependent canonical strain $\underline{\underline{\gamma }}$ applied to the system at t=0 under the condition of no flow at t>0 (ie no further strain is allowed, $\underline{\underline{\dot{\gamma }}}=0$ at t>0) indicated with the superscript ‘(r)’: [34,37]

(65) $$\Delta \sigma_{\alpha \beta }^{\left(r\right)}\left(\underline{q},t\right)=E_{\alpha \beta \gamma \delta }\left(\underline{q},t\right)\gamma_{\gamma \delta }\left(\underline{q}\right)$$

Here, $\underline{\underline{\gamma }}\left(\underline{r}\right)=\underline{\underline{\gamma }}\left(\underline{q}\right)e^{i\underline{q}\cdot \underline{r}}$ is defined by the particle displacement field (a particle located at $\underline{r}$ instantly moves to position $\underline{r}+\underline{u}\left(\underline{r}\right)$ at t=0)

(66) $$\underline{u}\left(\underline{r}\right)=\underline{u}\left(\underline{q}\right)e^{i\underline{q}\cdot \underline{r}}$$

Note that $\underline{q}$ is fixed here, so $\underline{u}\left(\underline{q}\right)$ and $\underline{\underline{\gamma }}\left(\underline{q}\right)$ are just a constant vector and tensor, respectively. Obviously (cf Equation (1)),

(67) $$\;\;\underline{\underline{\gamma }}\left(\underline{q}\right)\equiv i\underline{u}\left(\underline{q}\right)\underline{q}$$

To make the whole transformation canonical, a particle velocity $\underline{v}$ must also be changed as [37]

(68) $$\underline{v}\to \underline{v}-\underline{v}\cdot \underline{\underline{\gamma }}$$

The whole transformation, being canonical, conserves the phase space measure dΓ, and leads to the energy increment (cf Equation (19))

(69) $$\Delta H\left(\Gamma \right)=V\sigma_{\alpha \beta }\left(-\underline{q}\right)\gamma_{\alpha \beta }\left(\underline{q}\right)$$

Note that Equation (69) applies to each microstate Γ (the argument Γ being omitted in the rhs). The above equation can be verified, for example, using the microscopic definition of the *q*-dependent stress [57]

$$\sigma_{\alpha \beta }\left(\underline{q}\right)=\frac{1}{V}\int \sigma_{\alpha \beta }\left(\underline{r}\right)e^{-i\underline{q}\cdot \underline{r}}\;d^{d}r=$$

(70) $$=\frac{1}{V}\sum_{i>j}u_{ij}^{\prime }\left(r\right)\frac{r_{\alpha }r_{\beta }}{i\underline{q}\cdot \underline{r}}\frac{1}{r}\left(e^{-i\underline{q}\cdot {\underline{r}}_{i}}-e^{-i\underline{q}\cdot {\underline{r}}_{j}}\right)-\frac{1}{V}\sum_{i}m_{i}v_{i\alpha }v_{i\beta }e^{-i\underline{q}\cdot {\underline{r}}_{i}}$$

where the first sum includes all disordered pairs i,j of interacting particles (with positions ${\underline{r}}_{i}$, ${\underline{r}}_{j}$), $u_{ij}\left(r\right)$ is their interaction energy, $u_{ij}^{\prime }\left(r\right)=\frac{du_{ij}\left(r\right)}{dr}$, $\underline{r}={\underline{r}}_{j}-{\underline{r}}_{i}$, and $v_{i\alpha }$ is component α of the velocity of particle *i*. In the limit, q→0, Equation (70) agrees with Equation (18).

The first Equation (20), which is generally valid for transformations conserving dΓ, reads

(71) $$\Delta P\left(\Gamma \right)/P_{0}\left(\Gamma \right)=\frac{\Delta H(\Gamma )}{T}$$

Using it with Equation (69), we find that the perturbation of the system distribution in the phase space (due to the deformation, Equation (66)) is proportional to $\sigma_{\alpha \beta }\left(-\underline{q}\right)$.

Furthermore, from Equations (69) and (71), we deduce that if no external force is applied to the system at t>0 (ie the internal flow is allowed at t>0), the stress response $\Delta \underline{\underline{\sigma }}$ (to a weak instantaneous strain $\underline{\underline{\gamma }}$ at t=0) is provided by the stress correlation function defined in Equation (48):

(72) $$\Delta \sigma_{\alpha \beta }\left(\underline{q},t\right)=C_{\alpha \beta \gamma \delta }\left(\underline{q},t\right)\gamma_{\gamma \delta }\left(\underline{q}\right),\;\;t>0$$

(cf Equation (65)). The above equation was obtained in ref. [37] using slightly different notations (see Equations (6) and (7) there).

Importantly, exactly the same perturbation of P(Γ) can be achieved by application of an external force field, Equation (38), corresponding to the coherent acceleration field (cf Equations (40) and (43)) [32]

(73) $$A\left(\underline{r},t\right)=A\left(t\right)e^{i\underline{q}\cdot \underline{r}},\;\;A\left(t\right)=\underline{u}\left(\underline{q}\right)\dot{\delta }\left(t\right)$$

where $\dot{\delta }\left(t\right)=d\delta \left(t\right)/dt$ is the first derivative of Dirac’s δ:

$$\dot{\delta }\left(t\right)=\lim_{\Delta t\to 0}\frac{\delta (t)-\delta (t-\Delta t)}{\Delta t}$$

It is instructive to consider a finite Δt. Then, the effect of the external field can be viewed as a combination of a push (on each particle) at t=0 and the opposite push at t=Δt:

(74) $$A\left(\underline{r},t\right)=\underline{V}\left(\underline{q}\right)\left(\delta (t)-\delta (t-\Delta t)\right)e^{i\underline{q}\cdot \underline{r}},\;\;\underline{V}\left(\underline{q}\right)\equiv \underline{u}\left(\underline{q}\right)/\Delta t$$

The velocity increment due to the first push is $\Delta \underline{v}=\left(\underline{u}\left(\underline{q}\right)/\Delta t\right)e^{i\underline{q}\cdot \underline{r}}$, which leads to additional displacement $\Delta \underline{r}=\left(\Delta \underline{v}\right)\Delta t=\underline{u}\left(\underline{q}\right)e^{i\underline{q}\cdot \underline{r}}$ before braking, which agrees with Equation (66). Since without external forces, the dynamics is energy conserving, the energy change, ΔH=ΔH(Γ), comes solely from the two pushes, Equation (74). By virtue of Equation (44), the effect of the first push is

$$\Delta H_{1}=V\underline{J}\left(-\underline{q},0\right)\cdot \underline{V}\left(\underline{q}\right)$$

while the second (negative) push must lead to

$$\Delta H_{2}=-V\underline{J}\left(-\underline{q},\Delta t\right)\cdot \underline{V}\left(\underline{q}\right)$$

Taking into account both pushes, using Equation (49) and taking the limit Δt→0, we obtain

(75) $$\Delta H=\Delta H_{1}+\Delta H_{2}=Vu_{\alpha }\left(\underline{q}\right)iq_{\beta }\sigma_{\alpha \beta }\left(-\underline{q}\right)$$

which coincides with Equation (69). Hence, the effects of an instantaneous deformation (Equations (66) and (68)) and of an appropriate external acceleration (Equation (73)) for any ensemble-averaged quantity are exactly the same. (Note, however, that the particle velocity perturbations, $\Delta \underline{v}$, are different in the two cases: Equation (68) implies that $\Delta v_{\alpha }=-iv_{\beta }u_{\beta }\left(\underline{q}\right)q_{\alpha }e^{i\underline{q}\cdot \underline{r}}$, while from Equation (73), it follows that $\Delta v_{\alpha }=-iv_{\beta }u_{\alpha }\left(\underline{q}\right)q_{\beta }e^{i\underline{q}\cdot \underline{r}}$). This applies in particular to the stress response. The definitions of the elastic moduli in terms of the instantaneous canonical deformation and via the external force field (Equation (38)) are, therefore, totally equivalent. The latter definition can be applied also for an arbitrary time-dependence of the external acceleration field, $\underline{A}\left(\underline{r},t\right)$ (defining the external force, $\underline{f}\left(\underline{r},t\right)$, cf Equation (38)). Therefore, using the external force field as a perturbation appears to be a more versatile approach than that of Section 4.

Noteworthily, the above results are consistent with Equations (53) and (54) of ref. [37], providing another illustration of a close relationship between responses to a canonical deformation and to an external force of the type defined in Equation (38).

### 5.2. The Reduced Elasticity Tensor

Equation (59) looks like a general relationship between the *C*- and *E*-tensors of the forth rank. However, in fact it yields only three relations between the components of the two tensors. The main reason for this is that there are only three independent components of the elasticity tensor *E* for q≠0, while some of its components simply cannot be determined based on the standard definition (see Equation (65) or Equation (33)): the structure of the deformation tensor $\gamma_{\alpha \beta }\left(\underline{q}\right)=u_{\alpha }\left(\underline{q}\right)q_{\beta }$ (cf Equation (67)) is such that $E_{\alpha \beta \gamma \delta }\left(\underline{q},t\right)$ always comes in combination with $q_{\delta }$, ie as

(76) $$E_{\alpha \beta \gamma }\left(\underline{q},t\right)=E_{\alpha \beta \gamma \delta }\left(\underline{q},t\right)q_{\delta }$$

which is a third-rank tensor. That is why, for example, the component $E_{2222}$ (in the NRC) cannot be obtained. This is in contrast with the bulk case, q=0, where *all* components of the elasticity tensor $E_{\alpha \beta \gamma \delta }$ have physical significance and can be measured. It is natural to expect that the tensor $E_{\alpha \beta \gamma \delta }\left(\underline{q},t\right)$ at q→0 must coincide with the classical adiabatic moduli of the whole system (because the diffusive transport of heat, whose rate scales as $q^{2}$, vanishes for $\underline{q}\to 0$) [32,58]:

$$E_{\alpha \beta \gamma \delta }\left(\underline{q}\to 0,t\right)=E_{\alpha \beta \gamma \delta }^{\left(A\right)}\left(t\right)$$

On using Equations (24) and (61), we, therefore, find:

(77) $$M\left(q,t\right)\to M_{A}\left(t\right),\;\;G\left(q,t\right)\to G\left(t\right),\;\;L\left(q,t\right)\to M_{A}\left(t\right)+2G\left(t\right)\;\mathrm{as}q\to 0$$

where the subscript ‘*A*’ (and the superscript ‘(A)’) stand for ‘adiabatic’ (which is irrelevant for *G*, see text below Equation (24)).

Equation (65) can be written in terms of the tensor E as

(78) $$\Delta \sigma_{\alpha \beta }\left(\underline{q},t\right)=iE_{\alpha \beta \gamma }\left(\underline{q},t\right)u_{\gamma }\left(\underline{q}\right)$$

Since the displacement $u_{\gamma }$ can take any value (independently of $\underline{q}$), the above equation unambiguously define *all* components of the reduced elasticity tensor $E_{\alpha \beta \gamma }\left(\underline{q},t\right)$. Recalling the definitions of the material functions *G*, *L*, *M* (cf Equation (61)) and taking into account that $E_{\alpha \beta \gamma }\left(\underline{q},t\right)=E_{\beta \alpha \gamma }\left(\underline{q},t\right)$ is an isotropic tensor field, we find its unique expression in terms of the GRMs (cp Equation (11) of ref. [37]):

(79) $$E_{\alpha \beta \gamma }\left(\underline{q},s\right)=G\left(q,s\right)\left(q_{\alpha }\delta_{\beta \gamma }+q_{\beta }\delta_{\alpha \gamma }\right)+M\left(q,s\right)\delta_{\alpha \beta }q_{\gamma }\;+\left(L(q,s)-M(q,s)-2G(q,s)\right)q_{\alpha }q_{\beta }q_{\gamma }/q^{2}$$

Furthermore, Equation (59) can be rewritten in terms of the third-rank tensors as

(80) $$C_{\alpha \beta \gamma }\left(\underline{q},s\right)=E_{\alpha \beta \gamma }\left(\underline{q},s\right)-\frac{q_{\beta^{\prime }}}{\rho_{0}s^{2}}E_{\alpha \beta \alpha^{\prime }}\left(\underline{q},s\right)C_{\alpha^{\prime }\beta^{\prime }\gamma }\left(\underline{q},s\right)$$

where

(81) $$C_{\alpha \beta \gamma }\left(\underline{q},s\right)\equiv C_{\alpha \beta \gamma \delta }\left(\underline{q},s\right)q_{\delta }\;\;$$

Equation (80) allows to obtain all components of $C_{\alpha \beta \gamma }$ in terms of *s*-transforms of three VMFs (relaxation moduli): longitudinal, L(q,t), mixed/transverse, M(q,t), and shear, G(q,t).

To sum up, the elasticity tensor $E_{\alpha \beta \gamma \delta }\left(\underline{q},t\right)$ at q≠0 shows a sort of gauge invariance; its components can be varied without changing the material properties of the system, provided that the related (reduced) tensor $E_{\alpha \beta \gamma }\left(\underline{q},t\right)$ (cf Equation (76)) remains unchanged.

The inverse Fourier transform of $iE_{\alpha \beta \gamma }\left(\underline{q},t\right)$ is

(82) $${\tilde{E}}_{\alpha \beta \gamma }\left(\underline{r},t\right)=\frac{\partial }{\partial r_{\delta }}{\tilde{E}}_{\alpha \beta \gamma \delta }\left(\underline{r},t\right)$$

Indeed, Equation (29) implies that

$$iE_{\alpha \beta \gamma }\left(\underline{q},t\right)=\int {\tilde{E}}_{\alpha \beta \gamma }\left(\underline{r},t\right)\exp\left(-i\underline{q}\cdot \underline{r}\right)d^{d}r$$

and, hence, Equation (78) in real space becomes

(83) $$\Delta \sigma_{\alpha \beta }\left(\underline{r},t\right)=\int {\tilde{E}}_{\alpha \beta \gamma }\left(\underline{r}-{\underline{r}}^{\prime },t\right)u_{\gamma }\left({\underline{r}}^{\prime }\right)d^{d}r^{\prime }$$

For systems with short-range interactions, we consider (with interaction range ∼b, the molecular size) the function ${\tilde{E}}_{\alpha \beta \gamma }\left(\underline{r},t\right)$ must be localized within r≲b at t=0. For any t>0, the localization size increases in time, but remains finite. Hence, any integral over the real space involving ${\tilde{E}}_{\alpha \beta \gamma }\left(\underline{r},t\right)$ must converge. In particular, we obtain (recalling that a translation of the system as a whole does not lead to any stress):

(84) $$\int {\tilde{E}}_{\alpha \beta \gamma }\left(\underline{r},t\right)d^{d}r=0$$

Moreover, taking into account that for affine deformations, $\underline{u}$ is a linear function of $\underline{r}$, we find

(85) $$\int {\tilde{E}}_{\alpha \beta \gamma }\left(\underline{r},t\right)r_{\delta }d^{d}r=-E_{\alpha \beta \gamma \delta }\left(\underline{q}=0,t\right)=-E_{\alpha \beta \gamma \delta }^{\left(A\right)}\left(t\right)$$

ie the linear moments of ${\tilde{E}}_{\alpha \beta \gamma }\left(\underline{r},t\right)$ are related to the bulk relaxation moduli.

### 5.3. Why Asymmetric Strain?

Suppose the minor (γδ) symmetry of the elastic tensor holds: $E_{\alpha \beta \gamma \delta }\left(\underline{q},t\right)=E_{\alpha \beta \delta \gamma }\left(\underline{q},t\right)$. Then, the classical form of response (to the generalized $\underline{q}$-dependent canonical deformation) involving symmetrized strain $\epsilon_{\alpha \beta }=\left(\gamma_{\alpha \beta }+\gamma_{\beta \alpha }\right)/2$,

(86) $$\Delta \sigma_{\alpha \beta }\left(\underline{q},t\right)=E_{\alpha \beta \gamma \delta }\left(\underline{q},t\right)\epsilon_{\gamma \delta }$$

must give exactly the same stress response, $\Delta \sigma_{\alpha \beta }\left(\underline{q},t\right)$, as that given in Equation (65). However, while the (γδ) symmetry of *E* is guaranteed for q=0 (affine deformations), this generally may not be the case for nonzero *q*. Noteworthily, if the elasticity tensor shows a physically meaningful asymmetry, $E_{\alpha \beta \gamma \delta }-E_{\alpha \beta \delta \gamma }\neq 0$, then Equation (65) would capture the effect of this asymmetry, while Equation (86) would certainly miss it.

In fact, in terms of the classical affine deformation, a small shear along *x* with gradient along *y* is equivalent to a similar shear along *y* with gradient along *x* simply because the difference of these two shears is equivalent to a rotation of the system as a whole. The tensor $E_{\alpha \beta \gamma \delta }$ must, therefore, be symmetric with respect to permutation of γ and δ. However, such symmetry is not guaranteed in the case of $\underline{q}$-dependent deformations, where (for $\underline{q}$, say, parallel to the *x*-axis) a shear along *y* is possible, while a shear along *x* is not. Hence, the xy and yx shears at q≠0 can not be physically equivalent any more, and the strain symmetrization does not make sense.

The definition of the *E*-tensor with Equation (65) (and strain with Equation (1)) is, therefore, more general, and that is why it is used in the present paper.

## 6. Alternative Derivation of the C-E Relations Using the Concept of Stress Noise

### 6.1. Stress Noise and Flow-Induced Deterministic Stress

Following ref. [37], we split the instantaneous stress into two parts:

(87) $$\sigma_{\alpha \beta }\left(\underline{q},t\right)=\sigma_{\alpha \beta }^{n}\left(\underline{q},t\right)+\sigma_{\alpha \beta }^{d}\left(\underline{q},t\right)$$

where $\sigma^{d}$ is the flow-induced deterministic stress defined by the strain history (as in Equation (33)), while $\sigma^{n}$ is the ‘stress noise’ collecting all contributions to stress other than those caused by the strain history. The noise term $\sigma^{n}$ is omnipresent; it can be considered as a genuine stress fluctuation inherent in a system (or a system element) kept at zero strain. Such ‘random’ stress ($\sigma^{n}$) can be present even in an ideal gas due to temperature fluctuations, but can also stem from (possibly frozen) structural fluctuations (of local molecular packing) in the case of liquids and amorphous solids.

### 6.2. Deterministic and Noise Stresses with No Flow

Let us consider an ensemble of systems where the flow is arrested at *all times*. In what follows, we focus on a given (arbitrarily selected) wave-vector $\underline{q}$. So, the condition to be satisfied is ‘no flow’ at wave-vector $\underline{q}$:

(88) $$\underline{J}\left(\underline{q},t\right)=0$$

How to impose this condition given that the stress noise always tends to generate a fluctuative flow? The natural solution is to apply an appropriate external force field to the system (cf Equation (38)). The relevant external force must be harmonic in space:

(89) $${\underline{F}}_{i}\left(t\right)=m_{i}\underline{A}\left(t\right)e^{i\underline{q}\cdot {\underline{r}}_{i}\left(t\right)}+c.c.$$

where ${\underline{F}}_{i}$ is applied to particle *i* and *c.c.* stands for complex conjugate. (Note that the *c.c.* term is generally needed to keep the force real. It was omitted in Section 4 and Section 5 dealing with linear response since linearity implies additivity allowing to consider complex perturbations). The function $\underline{A}\left(t\right)$ provides a coherent ‘external’ acceleration of all particles. It must be chosen in such a way as to suppress the current $\underline{J}\left(\underline{q},t\right)$ in order to satisfy the condition (88) (cf Equation (36)) and thus suppressing also the strain rate (cf Equations (32) and (35)). Importantly, in this case, the total energy stays conserved in spite of the external force field, Equation (89).

The no-flow condition (88) does not imply that the deterministic stress $\sigma^{d}$ is absent, rather it ensures that $\sigma^{d}$ is time-independent for each system in the ensemble. In fact, the condition $\underline{J}\left(\underline{q},t\right)=0$ means (by virtue of mass conservation) that the mass density field at wave-vector $\underline{q}$ is frozen: $\rho \left(\underline{q},t\right)=\rho \left(\underline{q}\right)\;$ (cf Equation (39)). This frozen density fluctuation may be considered as having been created long ago as a result of a longitudinal deformation $\gamma_{\alpha \beta }\left(\underline{q}\right)=-\rho \left(\underline{q}\right){\hat{q}}_{\alpha }{\hat{q}}_{\beta }/\rho_{0}$ at t→−∞, where ${\hat{q}}_{\alpha }=q_{\alpha }/q$ is a unit vector in the direction of $\underline{q}$. (Naturally, one has to demand that just before the constraint, Equation (88), was imposed at t=−∞, the ensemble of systems was fully equilibrated to reach the canonical distribution in the phase space. Hence, the distribution of $\rho (\underline{q}$), being frozen, must remain canonical at all times). Such an initial deformation leads to a time-independent deterministic stress (for a given system)

(90) $$\sigma_{\alpha \beta }^{d}\left(\underline{q},t\right)=\sigma_{\alpha \beta }^{d}\left(\underline{q}\right)=-E_{\alpha \beta \gamma \delta }^{e}{\hat{q}}_{\gamma }{\hat{q}}_{\delta }\rho \left(\underline{q}\right)/\rho_{0}\;$$

where $E^{e}$ is the tensor of perfectly static (equilibrium) elastic moduli. As we consider a conceptually liquid regime (where all relaxation times are finite, albeit some of them may be extremely long), the equilibrium shear modulus $(G_{e}$) is zero, and (cf Equation (79))

(91) $$E_{\alpha \beta \gamma \delta }^{e}\left(\underline{q}\right){\hat{q}}_{\delta }=\left(L_{e}\left(q\right)-M_{e}\left(q\right)\right){\hat{q}}_{\alpha }{\hat{q}}_{\beta }{\hat{q}}_{\gamma }+M_{e}\left(q\right)\delta_{\alpha \beta }{\hat{q}}_{\gamma }$$

where $L_{e}\left(q\right)$ is the equilibrium longitudinal modulus (which is close to the bulk compression modulus in the liquid regime at low *q*), and $M_{e}\left(q\right)$ is the analogous mixed (transverse) modulus. The above equations lead to

(92) $$\sigma_{\alpha \beta }^{d}\left(\underline{q}\right)=-\kappa_{\alpha \beta }\rho \left(\underline{q}\right)/\rho_{0}$$

where

(93) $$\kappa_{\alpha \beta }\equiv \left(L_{e}\left(q\right)-M_{e}\left(q\right)\right){\hat{q}}_{\alpha }{\hat{q}}_{\beta }+M_{e}\left(q\right)\delta_{\alpha \beta }$$

Note that since the time-averaged stress noise, $\bar{\sigma^{n}}$, is always zero, $\sigma_{\alpha \beta }^{d}\left(\underline{q}\right)$ can also be interpreted as the time-averaged total stress, $\bar{\sigma_{\alpha \beta }\left(\underline{q},t\right)}$, for a given system with restricted dynamics, where Equation (88) is imposed for any *t*. In the above equation, we allow for a *q*-dependence of the equilibrium elastic moduli, although it is weak at low *q*, so that $L_{e}\left(q\right)≃L_{e}\left(0\right)$, and $M_{e}\left(q\right)≃M_{e}\left(0\right)$ (note also that $M_{e}\left(0\right)=L_{e}\left(0\right)$ and they both are equal to the equilibrium (static) bulk compression modulus since $G_{e}=0$ [37]). Therefore, taking also into account that all the material functions are even in *q*,

(94) $$L_{e}\left(q\right)-M_{e}\left(q\right)\propto q^{2}$$

The time-independent correlation function of $\sigma^{d}$,

(95) $$C_{\alpha \beta \gamma \delta }^{d(r)}\left(\underline{q}\right)\equiv \frac{V}{T}{\langle \sigma_{\alpha \beta }^{d}\left(\underline{q}\right)\sigma_{\gamma \delta }^{d}\left(-\underline{q}\right)\rangle }_{r}$$

is then simply defined by the density fluctuation and, hence, eventually by the equilibrium elastic moduli (the superscript ‘(r)’ and subscript ‘r’ mean with restricted dynamics). Using the generalized compressibility equation [57]

$$\langle {\left|\rho (\underline{q})\right|}^{2}\rangle /\rho_{0}^{2}=T/\left(VL_{e}\left(q\right)\right)$$

we then find that

(96) $$C_{\alpha \beta \gamma \delta }^{d(r)}\left(\underline{q}\right)=\kappa_{\alpha \beta }\kappa_{\gamma \delta }/L_{e}\left(q\right)$$

because ${\langle \rho \left(\underline{q}\right)\rho \left(-\underline{q}\right)\rangle }_{r}=\langle \rho \left(\underline{q}\right)\rho \left(-\underline{q}\right)\rangle$ since density fluctuations (with restricted dynamics) are frozen in at their value at t→−∞ when the system was fully equilibrated before the no-flow condition (88) was turned on.

By the concept introduced in ref. [37], the total stress is always a sum of the deterministic stress and noise, Equation (87), including the case of restricted dynamics, Equation (88). In the latter case $\sigma^{d}$ is constant (independent of time, cf Equation (90)), while the time-averaged stress noise, $\sigma_{\alpha \beta }^{n}\left(\underline{q},t\right)$, must vanish for each system (by virtue of its stochastic nature):

$$\bar{\sigma_{\alpha \beta }^{n}\left(\underline{q},t\right)}=0$$

This means that $\sigma^{n}$ and $\sigma^{d}$ are never correlated:

(97) $$\langle \sigma_{\alpha \beta }^{d}\left(\underline{q}\right)\sigma_{\gamma \delta }^{n}\left(-\underline{q},t\right)\rangle \equiv 0$$

and that the stress–noise correlation function

(98) $$C_{\alpha \beta \gamma \delta }^{n}\left(\underline{q},t\right)\equiv \frac{V}{T}\langle \sigma_{\alpha \beta }^{n}\left(\underline{q},t+t^{\prime }\right)\sigma_{\gamma \delta }^{n}\left(-\underline{q},t^{\prime }\right)\rangle$$

must vanish at t→∞. As a result, the correlation function of the total stress, $\sigma_{\alpha \beta }\left(\underline{q},t\right)=\sigma_{\alpha \beta }^{d}\left(\underline{q},t\right)+\sigma_{\alpha \beta }^{n}\left(\underline{q},t\right)$,

(99) $$C_{\alpha \beta \gamma \delta }^{\left(r\right)}\left(\underline{q},t\right)\equiv \frac{V}{T}{\langle \sigma_{\alpha \beta }\left(\underline{q},t+t^{\prime }\right)\sigma_{\gamma \delta }\left(-\underline{q},t^{\prime }\right)\rangle }_{r}$$

where ‘r’ indicates the restricted dynamics, becomes a sum of the deterministic (cf Equation (96)) and noise terms:

$$C_{\alpha \beta \gamma \delta }^{\left(r\right)}\left(\underline{q},t\right)=C_{\alpha \beta \gamma \delta }^{d(r)}\left(\underline{q}\right)+C_{\alpha \beta \gamma \delta }^{n}\left(\underline{q},t\right)$$

On the other hand, the total stress correlation function $C^{\left(r\right)}$ can be related to the stress-to-strain response using FDT just as is done in Section 5.1, so that Equation (72) becomes

(100) $$\Delta \sigma_{\alpha \beta }\left(\underline{q},t\right)=C_{\alpha \beta \gamma \delta }^{\left(r\right)}\left(\underline{q},t\right)\gamma_{\gamma \delta }\left(\underline{q}\right),\;\;t>0$$

Moreover, as the restricted dynamics do not allow for any further deformation (at wave-vector $\underline{q}$) for t>0, Equation (65) must be valid together with Equation (100):

(101) $$\Delta \sigma_{\alpha \beta }\left(\underline{q},t\right)=E_{\alpha \beta \gamma \delta }\left(\underline{q},t\right)\gamma_{\gamma \delta }\left(\underline{q}\right),\;t>0$$

The above two equations lead to

(102) $$C_{\alpha \beta \gamma \delta }^{\left(r\right)}\left(\underline{q},t\right)q_{\delta }=E_{\alpha \beta \gamma \delta }\left(\underline{q},t\right)q_{\delta },\;t>0$$

Equation (102), together with Equations (76) and (79), allow to obtain all the three material functions, G(q,t), L(q,t), M(q,t) from the stress correlation function $C^{\left(r\right)}$. Moreover, using simple properties of the stress-correlation functions stated above, we also obtain from Equation (102):

(103) $$C_{\alpha \beta \gamma \delta }^{d(r)}\left(\underline{q}\right)q_{\delta }=E_{\alpha \beta \gamma \delta }^{e}\left(\underline{q}\right)q_{\delta }$$

(as follows from Equations (91), (93) and (96)) and

(104) $$C_{\alpha \beta \gamma \delta }^{n}\left(\underline{q},t\right)q_{\delta }=\left[E_{\alpha \beta \gamma \delta }\left(\underline{q},\left|t\right|\right)-E_{\alpha \beta \gamma \delta }^{e}\left(\underline{q}\right)\right]q_{\delta }$$

Note that $\left|t\right|$ in the rhs renders the above equation valid also for t<0 due to time-reversibility of the restricted dynamics. A relation equivalent to Equation (104) was established (in a different form and using a different argument unrelated to the restricted dynamics) in ref. [37] (see Equation (51) there).

It is important that Equation (104) is general—the correlation properties of stress noise are the same no matter if external force is applied or not. Using Equation (104), together with the momentum equation for the classical (unconstrained) dynamics, one can (following the approach developed in ref. [37]) derive again Equation (59) for the stress correlation tensor and the three exact relations for its components, Equations (62)–(64). It shows that the concept of stress noise is consistent with the FDT-based approach developed in Section 4.

In the general case, Equation (59) can serve as a basis of the method to obtain all elastic moduli *E* in terms of stress correlation functions. Furthermore, due to Equation (102), this task become trivial with the constrained dynamics once $C^{\left(r\right)}$ is known. How about the reverse task: to obtain all components of *C* based on material functions? It may seem impossible since *C* generally involves five unknown invariant scalar functions (cf Equation (A16) in Appendix B), while *E* is characterized by only three well-defined functions, which naturally define just three independent components of the *C*-tensor (cf Equations (62)–(64)). Nevertheless, below (in Section 6.4), we consider an approximate way to obtain the remaining two equations in order to completely define the stress correlation tensor field *C*.

The fact that only three independent components of the *E*-tensor can be defined for q>0 based on the stress-to-strain response, Equation (65) (cf Section 5.2), means that we have some freedom in defining the other components of the *E*-tensor. The situation here is similar to the classical electrodynamics, where the electric and magnetic fields are measurable and are, therefore, unambiguously defined for a given system, while the scalar and vector (A) potentials (whose derivatives do define the physical fields) are not uniquely defined themselves, so there is a certain freedom of choosing A, which is known as gauge invariance. In this regard, an appropriate definition of the whole *E*-tensor can be based on a straightforward generalization of Equations (102)–(104) by postulating that

(105) $$E_{\alpha \beta \gamma \delta }\left(\underline{q},t\right)\equiv C_{\alpha \beta \gamma \delta }^{\left(r\right)}\left(\underline{q},t\right)=C_{\alpha \beta \gamma \delta }^{n}\left(\underline{q},t\right)+E_{\alpha \beta \gamma \delta }^{e}\left(\underline{q}\right)$$

where

(106) $$E_{\alpha \beta \gamma \delta }^{e}\left(\underline{q}\right)\equiv C_{\alpha \beta \gamma \delta }^{d(r)}\left(\underline{q}\right)=\kappa_{\alpha \beta }\kappa_{\gamma \delta }/L_{e}\left(q\right)$$

(cf Equation (96)) and $\kappa_{\alpha \beta }$ is defined in Equation (93). Obviously, this definition is consistent with Equations (102)–(104), and, therefore, it provides correct values of already defined components (like $E_{2121}=G\left(q,t\right)$). Moreover, $E_{\alpha \beta \gamma \delta }$ according to Equation (105) correctly tends to an isotropic tensor in the limit q→0 (cf Equation (7)) since (i) $C_{\alpha \beta \gamma \delta }^{n}$ always shows a finite correlation range and, therefore, must tend to an isotropic tensor for q→0 in Fourier space, and (ii) the factor $\kappa_{\alpha \beta }$ becomes isotropic as well, $\kappa_{\alpha \beta }\to M_{e}\delta_{\alpha \beta }$ (cf Equation (93)) as $L_{e}\left(q\right)-M_{e}\left(q\right)\to 0$ at q→0 (cf Equation (94)). Recall that all components of the *E*-tensor become measurable at q→0 and its general definition given above is necessarily correct in this limit (cf Equation (23) and note that $C=C^{\left(r\right)}$ at q=0). It is also obvious that $E_{\alpha \beta \gamma \delta }$ defined in Equation (105) must show all minor and major symmetries with respect to index permutations just like the bulk elasticity tensor (cf Equation (7)) because Equation (105) identifies $E_{\alpha \beta \gamma \delta }$ with the stress correlation function $C^{\left(r\right)}$ that does show all these symmetries for equilibrium isotropic systems (cf ref. [37]) since the restricted dynamics are time-reversible just like the classical dynamics.

### 6.3. No Flow at T<0: A Route to New Relations

For the purpose of argument, it is now convenient to consider the system where the flow at wave-vector $\underline{q}$ is arrested at t<0 (cf Equation (88)), but this constraint is released at t>0. In this case, $\sigma^{d}=const\;$ at t<0. After the constraint is released (external force suppressed at t>0), the system rapidly equilibrates to arrive at the genuine equilibrium distribution (with proper fluctuations of the current *J*). It may seem, therefore, that at short times, t>0, the stress correlation function defined as

(107) $$C_{\alpha \beta \alpha^{\prime }\beta^{\prime }}^{r0}\left(\underline{q},t\right)\equiv \frac{V}{T}{\langle \sigma_{\alpha \beta }\left(\underline{q},t\right)\sigma_{\alpha^{\prime }\beta^{\prime }}\left(-\underline{q},0\right)\rangle }_{r0}$$

with the constraint, $\underline{J}\left(\underline{q},t\right)=0$ at t<0 (indicated by the superscript ‘r0’) may be different from the genuine *equilibrium* correlation function of the total stress σ (obtained with the classical unconstrained dynamics)

(108) $$C_{\alpha \beta \alpha^{\prime }\beta^{\prime }}\left(\underline{q},t\right)\equiv \frac{V}{T}\langle \sigma_{\alpha \beta }\left(\underline{q},t+t^{\prime }\right)\sigma_{\alpha^{\prime }\beta^{\prime }}\left(-\underline{q},t^{\prime }\right)\rangle$$

The latter function does not depend on $t^{\prime }$ since the equilibrium state is obviously stationary. We show, however, that in fact

(109) $$C_{\alpha \beta \alpha^{\prime }\beta^{\prime }}^{r0}\left(\underline{q},t\right)=C_{\alpha \beta \alpha^{\prime }\beta^{\prime }}\left(\underline{q},t\right),\;\;t>0$$

(see Appendix A).

The total stress is always a sum of the deterministic stress $\sigma^{d}$ and the noise, $\sigma^{n}$, Equation (87). The deterministic stress at t=0, $\sigma_{\alpha \beta }^{d}\left(\underline{q},0\right)$, is never correlated with $\sigma^{n}$:

(110) $$\langle \sigma_{\alpha \beta }^{d}\left(\underline{q},0\right)\sigma_{\gamma \delta }^{n}\left(-\underline{q},t\right)\rangle =0$$

Indeed, for t≤0, the above equation simply follows from the results of the previous section (Equation (97)) since the release of the constraint (Equation (88)) at t=0 does not affect the system dynamics at t<0. On the other hand, the validity of Equation (110) at t>0 is hinged on the stochastic nature of the noise, $\sigma_{\alpha \beta }^{n}\left(\underline{q},t\right)$ (its independence of a weak flow in the linear regime). Recall now that the stress noise is omnipresent (both at t<0 and t>0) and its autocorrelation function is always related to the elasticity tensor, cf Equations (105) and (106):

(111) $$C_{\alpha \beta \gamma \delta }^{n}\left(\underline{q},t\right)=E_{\alpha \beta \gamma \delta }\left(\underline{q},\left|t\right|\right)-E_{\alpha \beta \gamma \delta }^{e}\left(\underline{q}\right)$$

The autocorrelation function of the deterministic stress at t=0, $\sigma_{\alpha \beta }^{d}\left(\underline{q},0\right)$, is defined by the rhs of Equation (96) (cf also Equations (92) and (93)) leading to (on recalling Equation (106)):

(112) $$\left(V/T\right)\langle \sigma_{\alpha \beta }^{d}\left(\underline{q},0\right)\sigma_{\gamma \delta }^{d}\left(-\underline{q},0\right)\rangle =E_{\alpha \beta \gamma \delta }^{e}\left(\underline{q}\right)$$

Turning to $\sigma_{\alpha \beta }^{d}\left(\underline{q},t\right)$ at t>0, based on the Boltzmann superposition principle, it must be a sum of $\sigma_{\alpha \beta }^{d}\left(\underline{q},t=0\right)$ and the contributions due to the flow at t>0. Thus, the total stress at t>0 is

(113) $$\sigma_{\alpha \beta }\left(\underline{q},t\right)=\sigma_{\alpha \beta }^{n}\left(\underline{q},t\right)+\sigma_{\alpha \beta }^{d}\left(\underline{q},0\right)+\int_{0}^{t}E_{\alpha \beta \gamma \delta }\left(\underline{q},t-t^{\prime }\right)iv_{\gamma }\left(\underline{q},t^{\prime }\right)q_{\delta }dt^{\prime }$$

where $\underline{v}=\underline{J}/\rho_{0}$ is the flow velocity $(\underline{J}$ is the mass current), and $iv_{\gamma }\left(\underline{q},t^{\prime }\right)q_{\delta }$ is the relevant strain rate at $t^{\prime }$ corresponding to $\partial v_{\gamma }/\partial r_{\delta }$ (cf Equation (33)). Note that the last term in Equation (113) is the time-dependent part of the deterministic stress, which is defined by the flow field history and the generalized elasticity tensor (cf Equations (27) and (33)).

Doing the *s*-transform (cf Equation (51)) of the above relation and taking into account the equation of motion (cf Equation (49))

$$\frac{\partial v_{\gamma }}{\partial t}=\frac{iq_{\gamma^{\prime }}}{\rho_{0}}\sigma_{\gamma \gamma^{\prime }}$$

we find

(114) $$\sigma_{\alpha \beta }\left(\underline{q},s\right)=\sigma_{\alpha \beta }^{n}\left(\underline{q},s\right)+\sigma_{\alpha \beta }^{d}\left(\underline{q},t=0\right)-\frac{q_{\delta }q_{\gamma^{\prime }}}{\rho_{0}s^{2}}E_{\alpha \beta \gamma \delta }\left(\underline{q},s\right)\sigma_{\gamma \gamma^{\prime }}\left(\underline{q},s\right)$$

Note that the term

$$\frac{iq_{\delta }}{s}E_{\alpha \beta \gamma \delta }\left(\underline{q},s\right)v_{\gamma }\left(\underline{q},t=0\right)$$

is omitted in the above equation since $v_{\gamma }\left(\underline{q},t=0\right)=0$ by preparation of the system. Next, multiplying Equation (114) with $\sigma_{\alpha^{\prime }\beta^{\prime }}\left(-\underline{q},t=0\right)=\sigma_{\alpha^{\prime }\beta^{\prime }}^{d}\left(-\underline{q},t=0\right)+\sigma_{\alpha^{\prime }\beta^{\prime }}^{n}\left(-\underline{q},t=0\right)$, we obtain using Equations (109)–(112):

(115) $$C_{\alpha \beta \alpha^{\prime }\beta^{\prime }}\left(\underline{q},s\right)=E_{\alpha \beta \alpha^{\prime }\beta^{\prime }}\left(\underline{q},s\right)-\frac{q_{\delta }q_{\gamma^{\prime }}}{\rho_{0}s^{2}}E_{\alpha \beta \gamma \delta }\left(\underline{q},s\right)C_{\gamma \gamma^{\prime }\alpha^{\prime }\beta^{\prime }}\left(\underline{q},s\right)$$

The above equation can be compared with Equation (45) of ref. [65]. The latter paper deals with overdamped systems of identical particles (with concentration *n*), where the friction forces overwhelm the inertia. In the overdamped regime, the term $\rho_{0}s^{2}$ must be replaced with $\zeta_{0}ns$. With this and other trivial reductions, Equation (115) becomes similar in structure to Equation (45) of ref. [65], suggesting that the irreducible memory kernel $M_{\alpha \beta \gamma \delta }$ (defined in Equation (22) of ref. [65]) is likely to correspond to the elasticity tensor $E_{\alpha \beta \gamma \delta }$. However, any rigorous proof of such a correspondence is missing at present (not to mention a significant effect of the overdamped dynamics on the relaxation moduli). This issue could be an interesting point for further study.

Noteworthily, the FDT relation, Equation (59), simply comes from Equation (115) after multiplying it by $q_{\beta^{\prime }}$. We tend to view Equation (115) as exact, just like Equation (59). Equation (115) allows to predict all components of the correlation tensor *C* based on the elasticity tensor *E*. First, obviously, Equation (115) leads to the already stated relations (62)–(64) defining three independent components of the *C*-tensor $(C_{G}$, $C_{L}$, $C_{M}$) in terms of measurable material functions (G, *L*, *M*) since these relations follow from Equation (59), which, in turn, follows from Equation (115). Second, Equation (115) also defines the remaining two independent components $C_{N}$ and $C_{P}$ (see Appendix B):

(116) $$C_{N}\left(q,s\right)=N\left(q,s\right)-\frac{M{\left(q,s\right)}^{2}}{\rho_{0}s^{2}/q^{2}+L\left(q,s\right)},\;C_{P}\left(q,s\right)=P\left(q,s\right)-\frac{M{\left(q,s\right)}^{2}}{\rho_{0}s^{2}/q^{2}+L\left(q,s\right)}$$

where $N\left(q,s\right)=E_{2222}\left(q,s\right)$, $P\left(q,s\right)=E_{2233}\left(q,s\right)$ using NRC.

We emphasize that the new functions N(q,s) and P(q,s) cannot be ‘measured’ directly based on the stress-to-strain response. Of course, they can be obtained (for example, in simulations) using their relation to the correlation function of stress noise, Equation (105). However, it would be better to try and obtain as much as possible from the measurable quantities (in particular, the *G*, *L* and *M* functions). This is performed in the next section, where we derive approximate versions of Equation (116) involving only the latter three material functions.

Noteworthily, in ergodic systems, all the stress correlation functions must vanish at t→∞; hence, in particular, $C_{N}\left(q,s\to 0\right)=0,$ $C_{P}\left(q,s\to 0\right)=0$, leading to

$$N\left(q,s\right)L\left(q,s\right)=M{\left(q,s\right)}^{2},\;N\left(q,s\right)=P\left(q,s\right),\;s\to 0$$

The above equations are valid in the liquid state $(T>T_{g}$). However, they are not valid any more in the glassy regime $(T<T_{g}$), unless we treat the condition s→0 literally (ie allowing for astronomical times *t*). In the glassy case, the transiently frozen stresses, $\sigma_{22}\left(\underline{q}\right)$, $\sigma_{33}(\underline{q)}$, $\sigma_{23}\left(\underline{q}\right)$, are generally present in the system for the experimentally accessible time-scales (cf ref. [32]). It is noteworthy, however, that other stress components, like $\sigma_{11}$ and $\sigma_{12}=\sigma_{21}$, never include any frozen part (see Discussion point 9 in ref. [32]), which is in line with the fact that the correlation functions $C_{G}$, $C_{L}$ and $C_{M}$ are all tending to 0 at low *s* even in glassy systems (cf Equations (62)–(64)). By contrast, the correlation functions involving only the ‘transverse’ stress components ($\sigma_{22}\left(\underline{q}\right)$, $\sigma_{33}(\underline{q)}$, $\sigma_{23}\left(\underline{q}\right)$) are generally nonzero in vitrified (amorphous) systems:

$$C_{N}\left(q,s\right)>0,C_{P}\left(q,s\right)\neq 0,\;C_{2323}\left(q,s\right)>0,\;s\ll 1/\tau_{s}$$

where the ‘2323’ component refers to the NRC, and $s\ll 1/\tau_{s}$ actually means the glassy plateau regime. The last condition also implies that $C_{N}\left(q,s\right)>C_{P}\left(q,s\right)$, as follows from the relation $2C_{2323}\left(q,s\right)=C_{N}\left(q,s\right)-C_{P}\left(q,s\right)=N\left(q,s\right)-P\left(q,s\right)$, which, in turn, comes from the general ‘isotropy’ relation, Equation (A16). The above inequalities reflect the presence of frozen stress fields in amorphous systems [44].

### 6.4. Approximate Relations between *C* and *E* Tensors

Let us consider the low-*q* regime, qb≪1. At the end of Section 6.2, we already argued that $E_{\alpha \beta \gamma \delta }\left(\underline{q},t\right)$ must become isotropic at q→0. To expand on this point now, let us turn to the last term, $E^{e}$ in Equation (105) defining $E_{\alpha \beta \gamma \delta }\left(\underline{q},t\right)$. According to Equation (106)

(117) $$E_{\alpha \beta \gamma \delta }^{e}\left(\underline{q}\right)=e_{0}\left(q\right)\delta_{\alpha \beta }\delta_{\gamma \delta }+e_{2}\left(q\right)\left(q_{\alpha }q_{\beta }\delta_{\gamma \delta }+q_{\gamma }q_{\delta }\delta_{\alpha \beta }\right)+e_{4}\left(q\right)q_{\alpha }q_{\beta }q_{\gamma }q_{\delta }$$

where

(118) $$e_{0}\left(q\right)=\frac{M_{e}{\left(q\right)}^{2}}{L_{e}\left(q\right)},\;e_{2}\left(q\right)=\frac{L_{e}\left(q\right)-M_{e}\left(q\right)}{q^{2}}\frac{M_{e}\left(q\right)}{L_{e}\left(q\right)},\;e_{4}\left(q\right)=\frac{{\left(L_{e}\left(q\right)-M_{e}\left(q\right)\right)}^{2}}{q^{4}L_{e}\left(q\right)}$$

The equilibrium (perfectly static) material functions $L_{e}\left(q\right)$, $M_{e}\left(q\right)$ must be continuous and analytical (at least near q=0) and, moreover, equal at q=0: $L_{e}\left(0\right)=M_{e}\left(0\right)$ since a liquid does not show any shear elasticity in the static regime $(G_{e}\left(q\right)=0$); in other words, its static stress response must be isotropic. As the characteristic length-scale of static elasticity is expected to be structural in nature (cf the paragraph below Equation (123)), it should be typically defined by the molecular size ∼b and/or the interaction range (assumed to be similar). Taking also into account that the material functions are even in *q*, we, therefore, expect that (cf Equation (94))

$$L_{e}\left(q\right)-M_{e}\left(q\right)∼L_{e}\left(0\right){\left(qb\right)}^{2},\;qb\ll 1$$

The above relation shows that all the prefactor functions $(e_{0}$, $e_{2}$, $e_{4}$) in Equation (117) are continuous and finite at q=0 and, hence, $E_{\alpha \beta \gamma \delta }^{e}\left(\underline{q}\right)$ is analytical as a function of *vector* $\underline{q}$. The main isotropic $e_{0}$-term in Equation (117) is $∼L_{e}\left(0\right)$, while the other two terms provide small corrections (for qb≪1): $e_{2}$-term is $\propto {\left(qb\right)}^{2}$, $e_{4}$-term is $\propto {\left(qb\right)}^{4}$.

A similar argument works for the stress noise correlation tensor $C_{\alpha \beta \gamma \delta }^{n}\left(\underline{q},t\right)$ involved in Equation (105). Considering the stress noise $\sigma^{n}$, it is convenient to assume that the flow is arrested (cf Equation (88)), so that the elements of the system are not deformed. Then, variations of $\sigma^{n}$ are primarily due to structural (molecular packing) fluctuations in the system, which are expected to be short-range (with correlation length ∼b). In this case, the correlation tensor $C_{\alpha \beta \gamma \delta }^{n}\left(\underline{q},t\right)$ would be nearly *q*-independent and, therefore, isotropic for qb≪1. There is, however, one complication—apart from structural fluctuations, $\sigma^{n}$ is also affected by fluctuations of conserved fields, like energy density and composition (in the widely encountered case of multi-component polydisperse systems) [32,66]. Importantly, these fields are scalar and, therefore, they mainly contribute to the isotropic part of $\sigma_{\alpha \beta }^{n}$ corresponding to pressure: $\delta \sigma_{\alpha \beta }^{n}=-\left(\delta p\right)\delta_{\alpha \beta }$, where δp is the pressure fluctuation due to temperature or composition variations. The eventual contribution of scalar fields to $C_{\alpha \beta \gamma \delta }^{n}\left(\underline{q},t\right)$ is, therefore, also mainly isotropic (proportional to $\delta_{\alpha \beta }\delta_{\gamma \delta }$). Anisotropic contributions come from the gradients of the conserved fields, which are small for qb≪1. The resultant structure of the correlation tensor $C_{\alpha \beta \gamma \delta }^{n}\left(\underline{q},t\right)$ (and of the elasticity tensor $E_{\alpha \beta \gamma \delta }\left(\underline{q},t\right)$ in view of Equation (105)) must, therefore, be similar to the rhs of Equation (117) involving the main isotropic term plus some quadratic and quartic terms of order of ${\left(qb\right)}^{2}$ and ${\left(qb\right)}^{4}$, respectively. Neglecting the latter terms (depending on the wave-vector orientation $\underline{\hat{q}}$), we arrive to the main approximation for the *E*-tensor at small qb:

(119) $$E_{\alpha \beta \gamma \delta }\left(\underline{q},t\right)≃a_{E}\left(q,t\right)\delta_{\alpha \beta }\delta_{\gamma \delta }+b_{E}\left(q,t\right)\left(\delta_{\alpha \gamma }\delta_{\beta \delta }+\delta_{\alpha \delta }\delta_{\beta \gamma }\right),\;qb\ll 1$$

(cf Equation (7)), where $a_{E}$ and $b_{E}$ must be identified as

(120) $$a_{E}\left(q,t\right)=L\left(q,t\right)-2G\left(q,t\right),\;b_{E}\left(q,t\right)=G\left(q,t\right)$$

in order to provide exact results for the shear and longitudinal moduli, $E_{2121}\left(q,t\right)=G\left(q,t\right)$ (note that G(q,t)≃G(t), the bulk shear relaxation modulus) and $E_{1111}\left(q,t\right)=L\left(q,t\right)$ using the NRC, cf Equation (61). Generally Equation (119) is valid up to a correction of $O(q^{2}b^{2})$. In particular, for the transverse modulus *M*, it gives:

(121) $$M\left(q,t\right)=L\left(q,t\right)-2G\left(q,t\right)+O\left(q^{2}b^{2}\right)$$

as already noted in ref. [32] (see also ref. [37]). Importantly, Equation (119) also yields the remaining two independent functions *N* and *P* (see text below Equation (116)):

(122) $$N\left(q,t\right)=L\left(q,t\right)+O\left(q^{2}b^{2}\right),\;P\left(q,t\right)=M\left(q,t\right)+O\left(q^{2}b^{2}\right)$$

All the components of the correlation tensor *C* can then be obtained based on G(q,t) and L(q,t) using Equations (62)–(64) and Equations (116). In particular, the above approximation, N(q,t)≃L(q,t), leads to

(123) $$C_{N}\left(q,s\right)≃L\left(q,s\right)-\frac{M{\left(q,s\right)}^{2}}{\rho_{0}s^{2}/q^{2}+L\left(q,s\right)}$$

which agrees with Equation (23) of ref. [37] (an equivalent equation was also stated as Equation (75) in ref. [32]). Note that Equation (23) was derived in ref. [37] for monodisperse systems with infinite thermal conductivity. However, as demonstrated above, it remains valid also for *polydisperse* systems with any thermal conductivity. Equation (123) was verified at qb≲0.5 in simulation studies on two-dimensional (2D) systems of polydisperse LJ particles [32,44]. In particular, Figures 3 and 4 of ref. [44] show that well below the glass transition temperature ($T_{g}$), the stress correlation function $C_{N}\left(q,t\right)$ is nearly constant for qb≲0.5 at long times *t*. In this regime, it is independent of *q* and *t*, $C_{N}\approx L-M^{2}/L\approx 4G\left(1-G/L\right)$, and is close to the Young’s modulus $e_{Y}=4G\left(1-G/L\right)$ for glassy 2D systems, cf refs. [37,44] (here, *G*,L and *M* are the long-time plateau values of the bulk moduli).

In a similar way, we obtain

(124) $$C_{P}\left(q,s\right)≃M\left(q,s\right)-\frac{M{\left(q,s\right)}^{2}}{\rho_{0}s^{2}/q^{2}+L\left(q,s\right)}$$

Note that Equation (124) is useful for three-dimensional systems, but is irrelevant in two dimensions, where $C_{P}\left(q,s\right)=C_{2233}\left(q,s\right)$ is not defined (cf Equation (A19)).

Noteworthily, at q→0 and t→0, the function $C_{N}\left(q,t\right)$ is related to the affine moduli, G(0) and M(0):

$$C_{N}\left(0,0\right)=M\left(0\right)+2G\left(0\right)$$

as follows from Equations (121), (122) and (116), which also lead to $\;C_{N}\left(0,0\right)=N\left(0,0\right)=L\left(0,0\right)=C_{L}\left(0,0\right)$.

At long *t*, the response of conserved fields to a local strain must relax; hence, the *q*-dependence of $a_{E}$ and $b_{E}$ at t→∞ should come solely from structural correlations (of molecular packing), which are short-range in liquids and glasses. As a result, the *q*-dependence of the elastic response (cf Equation (119)) can be neglected for qb≪1, ie the elastic response is expected to be essentially local at t→∞. This conclusion supports the argument presented below Equation (118).

Is it possible to improve on Equation (119) for the generalized elasticity tensor by obtaining, for example, the quadratic correction $∼O(q^{2}b^{2})$? This problem is tackled below, but only for 2D systems (d=2). In this case, the general expression for $E_{\alpha \beta \gamma \delta }\left(\underline{q},t\right)$ is given in Equation (A21) (see Appendix B). As discussed above, the terms depending on the wave-vector orientation $\underline{\hat{q}}$ must be small there:

$$c_{E}\left(q,t\right)\propto {\left(qb\right)}^{2},\;e_{E}\left(q,t\right)\propto {\left(qb\right)}^{4}$$

Therefore, in the quadratic approximation, we can neglect the last $e_{E}$-term:

$$E_{\alpha \beta \gamma \delta }\left(\underline{q},t\right)≃a_{E}\left(q,t\right)\delta_{\alpha \beta }\delta_{\gamma \delta }+b_{E}\left(q,t\right)\left(\delta_{\alpha \gamma }\delta_{\beta \delta }+\delta_{\alpha \delta }\delta_{\beta \gamma }\right)$$

$$+c_{E}\left(q,t\right)\left({\hat{q}}_{\alpha }{\hat{q}}_{\beta }\delta_{\gamma \delta }+{\hat{q}}_{\gamma }{\hat{q}}_{\delta }\delta_{\alpha \beta }\right)$$

Next, we can express the unknown functions $a_{E}$, $b_{E}$ and $c_{E}$ in terms of the measurable material functions *G*, *L* and *M* using Equations (61):

(125) $$a_{E}\left(q,t\right)=M\left(q,t\right)-\Delta L\left(q,t\right),\;b_{E}\left(q,t\right)=G\left(q,t\right),\;c_{E}\left(q,t\right)=\Delta L\left(q,t\right)$$

where

(126) ΔL(q,t)≡L(q,t)−M(q,t)−2G(q,t)

Therefore, we obtain

(127) N(q,t)≃L(q,t)−2ΔL(q,t)

which must be valid up to the ‘quartic’ correction $∼O(q^{4}b^{4})$. Equation (127) is in agreement with the first Equation (122) (improving it) since $\Delta L\left(q,t\right)\propto {\left(qb\right)}^{2}$, as follows from Equations (121) and (126). Interestingly, Equation (127) is also in agreement with equation

(128) $$N_{e}\left(q\right)=\frac{M_{e}{\left(q\right)}^{2}}{L_{e}\left(q\right)}$$

coming from Equations (117) and (118). Indeed, using Equations (126) and (127), taking into account that $G_{e}\left(q\right)=G\left(q,t\to \infty \right)=0$ and neglecting the $O(q^{4}b^{4})$ correction, we obtain $N_{e}\left(q\right)=N\left(q,t\to \infty \right)=L_{e}\left(q\right)-2\left(L_{e}\left(q\right)-M_{e}\left(q\right)\right)=2M_{e}\left(q\right)-L_{e}\left(q\right)$, which coincides (to $O(q^{4}b^{4})$) with Equation (128) since $L_{e}\left(q\right)-M_{e}\left(q\right)\propto {\left(qb\right)}^{2}$ (cf Equation (121)).

Noteworthily, Equation (128) can be written as

(129) $$N_{e}\left(q\right)=L_{e}\left(q\right)-2\Delta L_{e}\left(q\right)+{\left(\Delta L_{e}\left(q\right)\right)}^{2}/L_{e}\left(q\right)$$

where $\Delta L_{e}\left(q\right)$ is defined by Equation (126) for t→∞. The last term in the above equation comes from the quartic term in Equation (117), which is proportional to $q^{4}$ since generally $\Delta L\left(q,t\right)\propto q^{2}$. Generalizing Equation (129), we propose the following heuristic approximation

(130) $$N\left(q,s\right)≃L\left(q,s\right)-2\Delta L\left(q,s\right)+\frac{{\left(\Delta L(q,s)\right)}^{2}}{L(q,s)}\equiv \frac{{\left[M(q,s)+2G(q,s)\right]}^{2}}{L(q,s)}$$

which is supposed to include not only the quadratic $(q^{2}$) but also the quartic correction $(\propto q^{4}$) to the main approximation, Equation (122). The correlation function $C_{N}\left(q,t\right)$ can then be obtained more precisely using the first Equation (116), with *N* defined either in Equation (127) or Equation (130).

**Figure 1:**
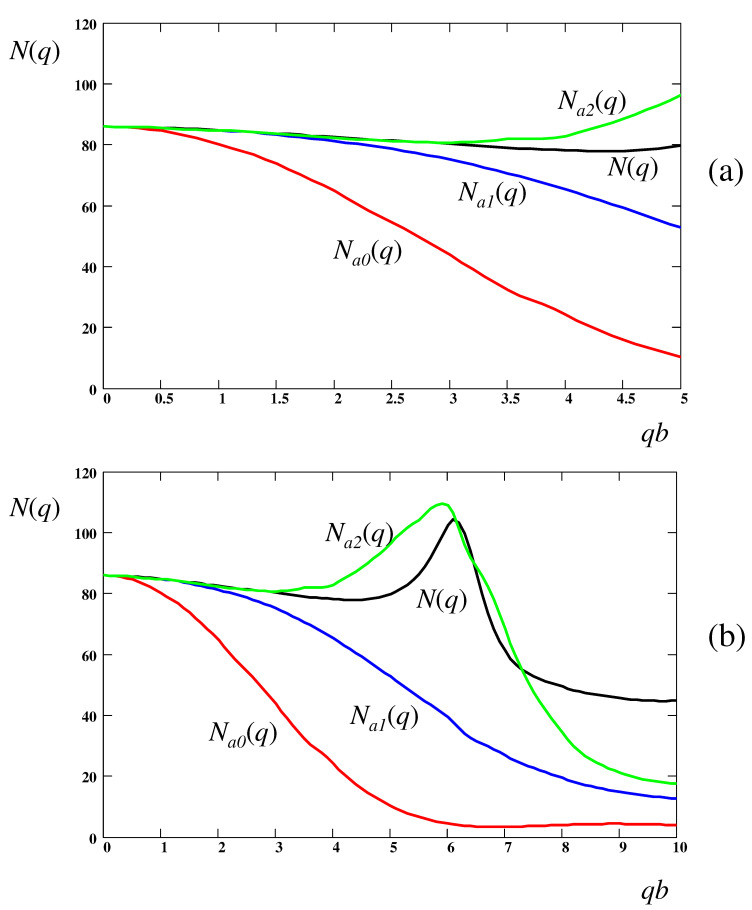
The wave-number (*q*) dependence of the instantaneous (adiabatic) elastic modulus N(q)≡N(q,t=0), black curve, and its three approximations based on the shear (G(q)), longitudinal (L(q)) and transverse (M(q)) elastic moduli: $N_{a0}\left(q\right)$, Equation (122) (red curve), $N_{a1}\left(q\right)$, Equation (127) (blue curve), and $N_{a2}\left(q\right)$, Equation (130) (green curve). All the moduli (N, *G*, *L*, *M*) are based on the stress correlation functions $C_{N}\left(q\right)$, $C_{G}\left(q\right)$, …obtained by MD simulations for a polydisperse system of LJ particles [32,58,60,66]. Panel (**a**) highlights the range 0<q<5, while panel (**b**) shows a wider range, 0<q<10, including the main structural peak at q≈6.3. Temperature T=0.4 (in LJ energy units) and the mean particle size b=1.

To verify the above predictions for N(q,t), we performed a simple test for t=0 (corresponding to s→∞) using simulation data for a 2D system of polydisperse LJ particles. This glass-forming system involving a weak polydispersity of the particle size (rather than mass) has been described elsewhere [32,58,60,66]. For s→∞, the basic Equation (115) gives:

$$C_{\alpha \beta \alpha^{\prime }\beta^{\prime }}\left(\underline{q},t=0\right)=E_{\alpha \beta \alpha^{\prime }\beta^{\prime }}\left(\underline{q},t=0\right)$$

leading to (the time argument t=0 is omitted):

$$G\left(q\right)=C_{G}\left(q\right),\;L\left(q\right)=C_{L}\left(q\right),\;M\left(q\right)=C_{M}\left(q\right),\;N\left(q\right)=C_{N}\left(q\right),$$

(Note that *G*, *L*, *M* here are the adiabatic moduli corresponding to the instantaneous stress response to an appropriate strain.) Thus, all the elastic material functions (G(q),…, N(q)) can be obtained directly based on the stress-correlation data from simulations. Figure 1 shows a comparison of the simulated N(q) with its approximations: 0th, $N_{a0}\left(q\right)$ based on N(q)=L(q), cf Equation (122); 1st, $N_{a1}\left(q\right)$ based on Equation (127); and second, $N_{a2}\left(q\right)$, from Equation (130). One can observe that the 0th approximation, $N_{a0}$, works well at qb<0.5, while $N_{a1}\left(q\right)$ and $N_{a2}\left(q\right)$ show excellent agreement with the simulated N(q) for qb<1.5 and qb<3, respectively (see Figure 1a). Thus, the second approximation allows to widen the *q*-region of applicability of the theory by a factor of 6. Moreover, the second approximation is also reasonable for larger *q*(qb>3), where it reproduces the peak of N(q) at qb∼6 in a qualitatively correct fashion, while the other two approximations show a qualitatively incorrect behavior at qb≳6 with $N_{a0}\left(q\right)$, showing a minimum instead of a peak there (cf Figure 1b).

## 7. Summary

1. In the present paper, we established and discussed a number of general relations between the 4th-rank tensor fields of stress correlations, $C_{\alpha \beta \alpha^{\prime }\beta^{\prime }}\left(\underline{q},t\right)$, cf Equation (48), and the tensor of generalized (viscoelastic) relaxation moduli, $E_{\alpha \beta \alpha^{\prime }\beta^{\prime }}\left(\underline{q},t\right)$, cf Equation (33). The *C*-tensor field is generally characterized by, at most, five independent components (invariant correlation functions, see Equation (A16) in Appendix B) as long as the minor and major symmetries of $C_{\alpha \beta \alpha^{\prime }\beta^{\prime }}$ are taken into account (cf Equations (A17)) [37]. By contrast, the *E*-tensor involves only three material functions, the generalized relaxation moduli (GRMs), G(q,t), L(q,t) and M(q,t) (cf Equations (61) and (79)), that can be measured according to their definition via stress response to a weak strain as given in Equation (33). It is, therefore, not surprising that there exist only three exact relationships (Equations (62)–(64)) linking the independent components of the *C*-tensor with the three material functions (since obviously five independent correlation functions cannot be expressed using only three material functions). Noteworthily, all the three GRMs can be obtained based on the correlation tensor using Equations (62)–(64) (which follow from the exact tensorial relation, Equation (59)). These three equations are rigorously derived in Section 4 based on the FDT. Equations (62) and (63) have been established before (cf refs. [34,37,57,59,63,64]. The last relation, Equation (64), was presented in ref. [37] and verified numerically in ref. [32]. It is also noteworthy that Equations (62) and (63) have been recently derived using the Zwanzig–Mori formalism [35].

2. In the case of affine deformations, the strain tensor is normally defined as the symmetric part of the tensor $\gamma_{\alpha \beta }$ of particle displacement gradients (cf Equations (1) and (4)). However, in the more general case of inhomogeneous deformations (which can be considered as a superposition of harmonic waves), the nonsymmetrized definition of strain, Equation (1), is more appropriate, as argued in Section 5.3.

3. We considered two definitions of the viscoelastic memory functions (VMFs): in terms of the stress response to a harmonic canonical strain (Equations (66)–(68)) and as a response to a coherent external acceleration field (Equations (73) and (38)). Importantly, it is demonstrated (see Section 5.1) that the two definitions lead to exactly the same response functions (G(q,t),L(q,t),M(q,t)). Remarkably, the approach involving the external force, Equation (38), appears to be more general than imposing a *q*-dependent canonical deformation: the latter can be reproduced with a singularity time-dependence of the external field, Equation (73).

4. It is also remarkable that the stress response to an arbitrary prescribed deformation of an amorphous system can be *completely* defined in terms of the reduced elasticity tensor, $E_{\alpha \beta \gamma }\left(\underline{q},t\right)$, introduced in Section 5.2 (cf Equation (76)). All components of this tensor can be obtained based on just three GRMs (= VMFs), G(q,t),L(q,t),M(q,t), cf Equation (79). The isotropic nature of the system dictates that these material functions are real and do not depend on the orientation of $\underline{q}$ (cf Equations (34), (76) and (79)). Moreover, as we argue in Section 6.4, these functions must be generally continuous and, moreover, analytical functions of $q^{2}$. At t=0, the elastic response is local. It is also likely that the same is virtually true at t→∞ (cf Section 6.4), so that, for example, $L\left(q,t\to \infty \right)≃L\left(0,t\to \infty \right)=M_{e}+2G_{e}$ at qb≪1 (with relative error $∼q^{2}b^{2}$, where *b* is the particle interaction range). Importantly, at low *q* (qb≪1), the three GRMs are related for any time *t* (cf Equation (121) and refs. [32,37]).

5. As mentioned above, in this study, we consider the elasticity tensor in terms of the stress response to a prescribed small strain or to an external force perturbation (in the latter case, the force generally depends on the particle position). Noteworthily, considering another type of perturbation by changing the system Hamiltonian from $H_{0}$ to $H=H_{0}+\Delta H$ with $\Delta H=-\epsilon_{\alpha \beta }\left(t\right)\sigma_{\alpha \beta }\left(\underline{q},t\right)$, involving a prescribed weak ‘deformation’ function $\epsilon_{\alpha \beta }\left(t\right)$, does not make much sense: On the one hand, it allows to employ the classical FDT [52], but on the other hand, it is unclear how the prescribed $\epsilon_{\alpha \beta }\left(t\right)$ can be possibly linked with the physical strain in the system given that the introduction of ΔH changes the classical relations between particle velocities and momenta leading to an anisotropic and position-dependent particle mass.

6. To uncover new relationships between the stress correlations and the elasticity tensor (cf Section 6), we employ the concept of the stress noise, $\sigma^{n}$, proposed in our previous paper [37]. It is defined as $\sigma^{n}\left(\underline{q},t\right)=\sigma \left(\underline{q},t\right)-\sigma^{d}\left(\underline{q},t\right)$, where $\sigma^{d}$ is the deterministic stress due to the flow history in the system (cf Equation (87)). The stress noise $\sigma^{n}$ can, thus, be considered as a genuine stress fluctuation unrelated to deformation and flow. This concept opened up the possibility to define *all components* of the generalized (q-dependent) elasticity tensor, $E_{\alpha \beta \gamma \delta }\left(\underline{q},t\right)$, in terms of the stress noise correlation function (cf Equations (98) and (105)). It is important that the new definition is totally consistent with the classical linear response way to introduce the elasticity tensor, Equation (33), and, therefore, leads to exactly the same GRMs, G(q,t),L(q,t),M(q,t). The latter statement is valid since Equations (59) and (62)–(64) trivially follow from Equation (115). On the other hand, the new definition, Equation (105), implies both minor and major symmetries of $E_{\alpha \beta \gamma \delta }\left(\underline{q},t\right)$, which are inherent in the classical *bulk* elasticity tensor. Moreover, the bulk tensor coincides with $E_{\alpha \beta \gamma \delta }\left(\underline{q},t\right)$ at q=0 since the latter tensor field is continuous and analytical as a function of $\underline{q}$ (see end of Section 6.2 and Section 6.4). The definition of the generalized elasticity tensor, Equation (105), therefore, combines the best of both worlds (of affine strains, q=0, and harmonic deformations, q≠0).

7. One may wonder how to obtain the correlation function of stress noise, $C_{\alpha \beta \gamma \delta }^{n}\left(\underline{q},t\right)$. The answer is given in Section 6.2: it can be done using simulations with arrested flow at wave-vector $\underline{q}$ implying the condition, Equation (88). This condition can be imposed using an external force field (cf Equation (89)) leading to an appropriate coherent harmonic acceleration of particles. With the constrained dynamics, the deterministic stress is always constant (time-independent); it is defined by the ‘quenched’ concentration fluctuation at $\underline{q}$. Then,

$$C_{\alpha \beta \gamma \delta }^{n}\left(\underline{q},t\right)=C_{\alpha \beta \gamma \delta }^{\left(r\right)}\left(\underline{q},t\right)-Const$$

where $C_{\alpha \beta \gamma \delta }^{\left(r\right)}\left(\underline{q},t\right)$ is the total stress correlation function with restricted dynamics (cf Equation (99)) and $Const\;=E_{\alpha \beta \gamma \delta }^{e}\left(\underline{q}\right)=C_{\alpha \beta \gamma \delta }^{d(r)}\left(\underline{q}\right)$ is a time-independent tensor, which, however, generally depends on $\underline{q}$ (cf Equations (96) and (106)). This tensor (Const) simply equals to $C_{\alpha \beta \gamma \delta }^{\left(r\right)}\left(\underline{q},t\to \infty \right)$; it is related to the equilibrium elastic moduli (at t→∞), cf Equations (92) and (95).

8. In Section 6.2, we introduced the equilibrium elasticity tensor $E_{\alpha \beta \gamma \delta }^{e}\left(\underline{q}\right)$ defined in Equation (106). In the liquid regime, $E_{\alpha \beta \gamma \delta }^{e}\left(\underline{q}\right)$ coincides with the static elasticity tensor, $E_{\alpha \beta \gamma \delta }^{e}\left(\underline{q}\right)=E_{\alpha \beta \gamma \delta }\left(\underline{q},t\gg \tau_{s}\right)$, so that $E_{\alpha \beta \gamma \delta }^{e}\left(\underline{q}\right)$ can be considered as a generalization of the classical static elasticity tensor (cf Section 2) for nonzero $\underline{q}$. However, in the glassy (amorphous solid) state, the two tensors, equilibrium and static, are different since even a very long waiting time, $t\gg \tau_{s}$, may not ensure a complete equilibration of a vitrified liquid (amorphous solid). In particular, the stress noise may include a virtually frozen component leading to an incomplete relaxation. Therefore, the static shear modulus, $G(q,t\gg \tau_{s}$), remains finite in this case, while the analogous *equilibrium* shear modulus must vanish since a *complete* equilibration after a small shear deformation of a glassy system must relax the shear stress due to the amorphous structure of the system [37]. (Note that we do not consider here a *permanently* crosslinked network whose equilibrium shear modulus is, of course, finite.) As a result, the equilibrium elasticity tensor can be expressed in terms of just two material functions: the equilibrium longitudinal, $L_{e}\left(q\right)$, and transverse, $M_{e}\left(q\right)$, elastic moduli (cf Equation (106)). These moduli, by their definition, provide a linear stress response (after a complete relaxation of the system) to a weak imposed longitudinal strain.

9. The most general relation between the stress-correlation (C) and elasticity (E) tensors is given in Equation (115). It is noteworthy that this equation was derived and is valid at q≠0. It cannot be generally applied for q=0 since the stress-correlation function *C* is ensemble-dependent in this case [32,37]. It is also remarkable that, based on Equation (115), we not only arrive at Equations (62)–(64) linking the shear, longitudinal and transverse components of *C*- and *E*-tensors, but also obtain two additional exact relations (116) involving other components of these tensors. The whole set of these relations then allows to obtain all components of the correlation tensor in terms of the elasticity tensor and vice versa. Strictly speaking, all the relations, Equations (62)–(64) and (116), are valid both for liquid systems (above the glass transition) and for amorphous solids (vitrified liquids), provided that they are completely equilibrated thermodynamically (this condition refers to the fact that the derivation of these relations assumed an equilibrium ensemble). Nevertheless, these relations are also valid for metastable glassy systems (trapped in a metabasin), provided that the lifetime of the metastable state is much longer than 1/s [32] and with the reservation that some $\underline{q}$-dependent constants may have to be added in the rhs of Equations (116), cf ref. [32]. These constants are due to the presence of frozen stresses in glassy systems, reflecting their metastable nature (within a given metabasin); they must disappear upon averaging over the full equilibrium ensemble of metastable states.

10. There is a subtle problem associated with the new Equations (116): they involve two new memory functions, N(q,t) and P(q,t), which cannot be obtained based on the stress response to a deformation, and, therefore, apparently cannot be measured experimentally. One may wonder if these functions can be obtained based on the ‘classical’ relaxation moduli, G(q,t),L(q,t),M(q,t). Our view is that while their exact prediction is generally impossible, the new functions *N* and *P* can be still predicted approximately at low *q*. As argued in Section 6 (see end of Section 6.2 and the beginning of Section 6.4), the elasticity tensor becomes nearly isotropic at low *q*, so that N(q,t)≃L(q,t) and P(q,t)≃M(q,t) at qb≪1 (cf Equations (122)). Replacing *N* with *L* in the first Equation (116) leads to an approximate equation, which was derived and rather thoroughly tested in refs. [32,37] using simulation data for a 2D system of polydisperse LJ particles. A very good agreement (with an accuracy of 1–2%) between $C_{N}$ and its approximate prediction was observed at qb≲0.5 (where *b* is the interaction range) [32]. Here, we devised two more precise approximations for N(q,t) valid for 2D systems (see Equations (127) and (130)). All the approximations have been tested at t=0 for a wide range of *q* for the same system. The comparison (between the exact and approximate N(q)=N(q,0)) is shown in Figure 1. It demonstrates that the basic (0th) approximations still work for qb<0.5, while the new approximations are accurate in much wider *q*-regions: the first one is valid at qb<1.5, the second at qb<3.

11. To summarize, let us highlight the main new results presented in the paper:

(i) We provide a rigorous derivation of Equations (63) and (64) using FDT-based arguments (cf Section 4). These equations have been previously stated in refs. [32,37], but their detailed derivation was not worked out (note that Equation (63) was also stated in ref. [59]). Importantly, in Section 4, we provide a derivation of the unique fully tensorial equation (Equation (59)) from which the general FDT relations, Equation (62) (which is well-known [57,63,64]) and Equations (63) and (64), simply follow in a trivial way.

(ii) We derived approximate Equations (123) and (124) (valid for qb≪1) using FDT and the concept of stress noise (cf Section 6.3). Note that the derivation of Equation (123) was only hinted at previously (in ref. [37]), while the same equation was simply claimed in ref. [32].

(iii) Building upon the concept of stress noise, a key result of our work is Equation (115), relating the tensor of stress correlations $C_{\alpha \beta \gamma \delta }\left(\vec{q},t\right)$ with the tensor of elastic moduli $E_{\alpha \beta \gamma \delta }\left(\vec{q},t\right)$. Noteworthily, the form of Equation (115) agrees with Equation (45) of ref. [49], which establishes a relation between the memory kernel $M_{\alpha \beta \gamma \delta }\left(\vec{q},t\right)$ from the Zwanzig–Mori projection operator formalism and $C_{\alpha \beta \gamma \delta }\left(\vec{q},t\right)$ for monodisperse Brownian particles. This suggests the intriguing possibility of a deeper connection between $M_{\alpha \beta \gamma \delta }\left(\vec{q},t\right)$ and $E_{\alpha \beta \gamma \delta }\left(\vec{q},t\right)$, which is an interesting topic for future studies.

(iv) For 2D systems, we, for the first time, derived more precise equations (as compared to Equation (123)) defining the stress correlation function $C_{N}\left(q,t\right)$ in terms of the generalized relaxation moduli (cf the first Equation (116) and Equations (127) and (130)).

## Data Availability

The original contributions presented in the study are included in the article, further inquiries can be directed to the corresponding author.

## Appendix A. Proof of Equation (109)

To simplify notations, let us define any component of the stress tensor at t=0 (taking either Re or Im part of it) as *X*, $X=\left(ℜ|ℑ\right)\sigma_{\alpha^{\prime }\beta^{\prime }}\left(\underline{q},0\right)$, and similarly, *Y* corresponds to a later moment *t*: $Y=\left(ℜ|ℑ\right)\sigma_{\alpha \beta }\left(\underline{q},t\right)$, while *Z* is defined as a component of the current $\underline{J}$ at t=0: $Z=\left(ℜ|ℑ\right)J_{\alpha }\left(\underline{q},0\right)$. More precisely, let us consider *Z* as a vector with 2d components, $\underline{Z}=\left\{Z_{i},i=1.2d\right\}$, where *d* is the space dimension. Next, we observe that (i) *X*, *Y* and $Z_{i}$ are collective variables (fluctuation amplitudes), so their joint distribution must be nearly Gaussian (with high accuracy, relative error ∼1/N), (ii) for $\underline{q}\neq 0$

(A1) $$\langle X\rangle =\langle Y\rangle =\langle Z_{i}\rangle =0$$

and (iii) *X* and $Z_{i}$ are uncorrelated at equilibrium,

(A2) $$\langle XZ_{i}\rangle =0$$

To justify the last statement, it is enough to recall the time reversibility of the dynamics and to note that *X* is invariant, while $Z_{i}$ changes sign once the time is reversed, t→−t.

The correlation functions in Equations (107) and (108) can be considered as linear combinations of terms like ${\langle XY\rangle }_{r}$ (for Equation (107)) and $\langle XY\rangle$ (for Equation (108)). Therefore, to prove the validity of Equation (109), it is enough to show that

(A3) $${\langle XY\rangle }_{r}=\langle XY\rangle$$

for all components *X* and *Y*. Here, $\langle XY\rangle$ means the unconstrained average of XY (with totally equilibrium state at t=0), and

(A4) $${\langle XY\rangle }_{r}\equiv {\langle XY\rangle }_{\underline{Z}=0}$$

implies the averaging under the condition that the initial state (at t=0) was prepared with the restricted dynamics, which boils down to the condition $\underline{Z}=0$.

Let us treat the rhs of Equation (A3). It is useful to represent the unconstrained joint distribution density of $\left(X,Y,\underline{Z}\right)$ as

(A5) $$\rho \left(X,Y,\underline{Z}\right)=\rho \left(X\right)\rho \left(\underline{Z}|X\right)\rho \left(Y|X,\underline{Z}\right)$$

where ρ(X) is the distribution density of *X* considered separately, $\rho (\underline{Z}|X)$ is the conditional distribution of $\underline{Z}$ for a given value of the variable *X*, and $\rho (Y|X,\underline{Z})$ is a similar conditional distribution of *Y* for the given *X* and $\underline{Z}$ (note that the ρ-functions are distinguished according to their variables). Thus, we obtain

(A6) $$\langle XY\rangle =\int XY\rho \left(X,Y,\underline{Z}\right)dXdYd^{2d}Z=\int X{\langle Y\rangle }_{X}\rho \left(X\right)dX$$

where

(A7) $${\langle Y\rangle }_{X}=\int {\langle Y\rangle }_{X,\underline{Z}}\rho \left(\underline{Z}|X\right)d^{2d}Z,\;{\langle Y\rangle }_{X,\underline{Z}}=\int Y\rho \left(Y|X,\underline{Z}\right)dY\;$$

are the mean values of *Y* for a given *X*, and for the given *X* and $\underline{Z}$, respectively. Introducing $\rho (X,Y|\underline{Z}$) as the conditional probability distribution of *X*,Y for a prescribed $\underline{Z}$, and similarly $\rho (X|\underline{Z}$), one finds

(A8) $${\langle XY\rangle }_{r}=\int XY\rho \left(X,Y|\underline{Z}=0\right)dXdY=\int X\rho \left(X|\underline{Z}=0\right){\langle Y\rangle }_{X,\underline{Z}=0}dX$$

Here, all the probability distribution (ρ-) functions are Gaussian. The following obvious relation is useful:

(A9) $${\langle Y\rangle }_{X,\underline{Z}}=\alpha X+\beta_{i}Z_{i}$$

as follows from Equation (A1) and the Gaussian nature of the distributions (α and $\beta_{i}$ are some constants; the sum over *i* is assumed). In a similar way, we find (cf Equations (A7) and (A9)):

(A10) $${\langle Y\rangle }_{X}=\alpha X+\beta_{i}{\langle Z_{i}\rangle }_{X}$$

and

(A11) $${\langle Z_{i}\rangle }_{X}=\gamma_{i}X$$

Finally, noting that

(A12) $$\langle XZ_{i}\rangle \equiv \langle X{\langle Z_{i}\rangle }_{X}\rangle =\gamma_{i}\langle X^{2}\rangle$$

and using Equation (A2), we find $\gamma_{i}=0$, so that ${\langle Y\rangle }_{X}=\alpha X$ and

(A13) $$\langle XY\rangle =\langle X{\langle Y\rangle }_{X}\rangle =\alpha \langle X^{2}\rangle$$

Turning to the lhs of Equations (A3) and (A4), corresponding to the case with constraint at t=0, in order to obtain ${\langle XY\rangle }_{r}$, we must impose the condition $\underline{Z}=0$ in Equation (A9) leading immediately to ${\langle Y\rangle }_{X,\underline{Z}=0}=\alpha X$ and, therefore, to (cf Equation (A8))

(A14) $${\langle XY\rangle }_{r}={\langle X{\langle Y\rangle }_{X,\underline{Z}=0}\rangle }_{r}=\alpha \int X^{2}\rho \left(X|\underline{Z}=0\right)dX$$

Now, we again take into account that *X* and $\underline{Z}$ are not correlated (Equation (A2)); hence, $\rho (X|\underline{Z})$ is independent of $\underline{Z}$, $\rho \left(X|\underline{Z}\right)=\rho \left(X\right)$. Therefore, we obtain (cf (A14))

(A15) $${\langle XY\rangle }_{r}=\alpha \langle X^{2}\rangle$$

Equations (A13) and (A15) directly lead to Equation (A3) and, therefore, to Equation (109).

## Appendix B. The Relevant Properties of Isotropic Tensor Fields

Tensor fields (like $C_{\alpha \beta \gamma \delta }\left(\underline{q},t\right)$) characterizing isotropic systems are isotropic in the sense that they are invariant with respect to a simultaneous rotation of the coordinate frame and the vector argument $\underline{q}$ [44]. Such isotropic tensor functions can be written in the general case as [35,37,41,44] (here $\underline{\hat{q}}\equiv \underline{q}/q$, $q\equiv \left|\underline{q}\right|\neq 0$):

(A16) $$C_{\alpha \beta \gamma \delta }\left(\underline{q},t\right)=a\left(q,t\right)\delta_{\alpha \beta }\delta_{\gamma \delta }+b\left(q,t\right)\left(\delta_{\alpha \gamma }\delta_{\beta \delta }+\delta_{\alpha \delta }\delta_{\beta \gamma }\right)+c\left(q,t\right)\left({\hat{q}}_{\alpha }{\hat{q}}_{\beta }\delta_{\gamma \delta }+{\hat{q}}_{\gamma }{\hat{q}}_{\delta }\delta_{\alpha \beta }\right)\;+d\left(q,t\right)\left({\hat{q}}_{\alpha }{\hat{q}}_{\gamma }\delta_{\beta \delta }+{\hat{q}}_{\alpha }{\hat{q}}_{\delta }\delta_{\beta \gamma }+{\hat{q}}_{\beta }{\hat{q}}_{\gamma }\delta_{\alpha \delta }+{\hat{q}}_{\beta }{\hat{q}}_{\delta }\delta_{\alpha \gamma }\right)+e\left(q,t\right){\hat{q}}_{\alpha }{\hat{q}}_{\beta }{\hat{q}}_{\gamma }{\hat{q}}_{\delta }$$

provided that (for any $\underline{q},t$), the *C*-tensor obeys both minor and major symmetries:

(A17) $$C_{\alpha \beta \gamma \delta }=C_{\beta \alpha \gamma \delta },\;C_{\alpha \beta \gamma \delta }=C_{\alpha \beta \delta \gamma },\;C_{\alpha \beta \gamma \delta }=C_{\gamma \delta \alpha \beta }$$

The first two (minor) symmetries obviously apply to the stress-correlation tensor (cf Equation (48)): they come from the well-known symmetry of the stress tensor, $\sigma_{\alpha \beta }=\sigma_{\beta \alpha }$. The last (major) symmetry comes from the time-reversibility of the dynamics, the isotropy of the tensor field $C_{\alpha \beta \gamma \delta }\left(\underline{q},t\right)$ and its minor symmetries (cf ref. [37]). The same symmetries also apply to the tensor of generalized elastic constants (according to Equation (105)).

For $\underline{q}$ parallel to the *x*-axis, there is no need to rotate the coordinate frame: it is already ‘natural’ in this case (ie it coincides with the NRC frame). Then, using Equation (A16) for such $\underline{q}$, we obtain:

$$a\left(q,t\right)=C_{P}\left(q,t\right),\;b\left(q,t\right)=\left(C_{N}-C_{P}\right)/2,\;c\left(q,t\right)=C_{M}-C_{P},$$

(A18) $$d\left(q,t\right)=C_{G}-\left(C_{N}-C_{P}\right)/2,\;e\left(q,t\right)=C_{L}+C_{N}-2C_{M}-4C_{G}$$

where

(A19) $$C_{N}\left(q,t\right)=C_{2222}\left(q,t\right),C_{P}\left(q,t\right)=C_{2233}\left(q,t\right)$$

and the arguments (q,t) are omitted in the rhs.

For 2D systems (d=2), the *a*, *b*, *c* and *d* terms of the general Equation (A16) become entangled due to the mathematical identity

(A20) $${\hat{q}}_{\alpha }{\hat{q}}_{\gamma }\delta_{\beta \delta }+{\hat{q}}_{\alpha }{\hat{q}}_{\delta }\delta_{\beta \gamma }+{\hat{q}}_{\beta }{\hat{q}}_{\gamma }\delta_{\alpha \delta }+{\hat{q}}_{\beta }{\hat{q}}_{\delta }\delta_{\alpha \gamma }=\;2\left({\hat{q}}_{\alpha }{\hat{q}}_{\beta }\delta_{\gamma \delta }+{\hat{q}}_{\gamma }{\hat{q}}_{\delta }\delta_{\alpha \beta }\right)-2\delta_{\alpha \beta }\delta_{\gamma \delta }+\left(\delta_{\alpha \gamma }\delta_{\beta \delta }+\delta_{\alpha \delta }\delta_{\beta \gamma }\right),\;d=2$$

As a result, Equation (A16) can be simplified as

(A21) $$E_{\alpha \beta \gamma \delta }\left(\underline{q},t\right)=a_{E}\left(q,t\right)\delta_{\alpha \beta }\delta_{\gamma \delta }+b_{E}\left(q,t\right)\left(\delta_{\alpha \gamma }\delta_{\beta \delta }+\delta_{\alpha \delta }\delta_{\beta \gamma }\right)\;+c_{E}\left(q,t\right)\left({\hat{q}}_{\alpha }{\hat{q}}_{\beta }\delta_{\gamma \delta }+{\hat{q}}_{\gamma }{\hat{q}}_{\delta }\delta_{\alpha \beta }\right)+e_{E}\left(q,t\right){\hat{q}}_{\alpha }{\hat{q}}_{\beta }{\hat{q}}_{\gamma }{\hat{q}}_{\delta },\;d=2$$

where we replaced *C* with *E*, *a* with $a_{E}$, etc.
